# Disrupting Mitochondrial Pyruvate Uptake Directs Glutamine into the TCA Cycle away from Glutathione Synthesis and Impairs Hepatocellular Tumorigenesis

**DOI:** 10.1016/j.celrep.2019.07.098

**Published:** 2019-09-03

**Authors:** Sean C. Tompkins, Ryan D. Sheldon, Adam J. Rauckhorst, Maria F. Noterman, Shane R. Solst, Jane L. Buchanan, Kranti A. Mapuskar, Alvin D. Pewa, Lawrence R. Gray, Lalita Oonthonpan, Arpit Sharma, Diego A. Scerbo, Adam J. Dupuy, Douglas R. Spitz, Eric B. Taylor

**Affiliations:** 1 Department of Biochemistry, University of Iowa Carver College of Medicine, Iowa City, IA 52240, USA; 2 Free Radical and Radiation Biology Program, Department of Radiation Oncology, University of Iowa Carver College of Medicine, Iowa City, IA 52240, USA; 3 Department of Anatomy and Cell Biology, University of Iowa Carver College of Medicine, Iowa City, IA 52240, USA; 4 Holden Comprehensive Cancer Center, University of Iowa Carver College of Medicine, Iowa City, IA 52240, USA; 5 Fraternal Order of Eagles Diabetes Research Center (FOEDRC), University of Iowa Carver College of Medicine, Iowa City, IA 52240, USA; 6 Abboud Cardiovascular Research Center, University of Iowa Carver College of Medicine, Iowa City, IA 52240, USA; 7 Pappajohn Biomedical Institute, University of Iowa Carver College of Medicine, Iowa City, IA 52240, USA; 8 FOEDRC Metabolomics Core Research Facility, University of Iowa Carver College of Medicine, Iowa City, IA 52240, USA; 9 These authors contributed equally; 10 Lead Contact

## Abstract

Hepatocellular carcinoma (HCC) is a devastating cancer increasingly caused by non-alcoholic fatty liver disease (NAFLD). Disrupting the liver Mitochondrial Pyruvate Carrier (MPC) in mice attenuates NAFLD. Thus, we considered whether liver MPC disruption also prevents HCC. Here, we use the N-nitrosodiethylamine plus carbon tetrachloride model of HCC development to test how liver-specific MPC knock out affects hepatocellular tumorigenesis. Our data show that liver MPC ablation markedly decreases tumorigenesis and that MPC-deficient tumors transcriptomically downregulate glutathione metabolism. We observe that MPC disruption and glutathione depletion in cultured hepatomas are synthetically lethal. Stable isotope tracing shows that hepatocyte MPC disruption reroutes glutamine from glutathione synthesis into the tricarboxylic acid (TCA) cycle. These results support a model where inducing metabolic competition for glutamine by MPC disruption impairs hepatocellular tumorigenesis by limiting glutathione synthesis. These findings raise the possibility that combining MPC disruption and glutathione stress may be therapeutically useful in HCC and additional cancers.

## In Brief

Tompkins et al. utilize stable glutamine isotope tracers *in vivo* and *ex vivo* to demonstrate hepatocyte MPC disruption increases TCA cycle glutamine utilization at the expense of glutathione synthesis and decreases hepatocellular tumorigenesis.

## Graphical Abstract

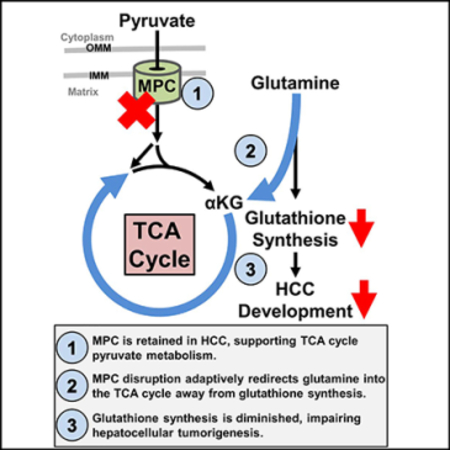

## INTRODUCTION

Hepatocellular carcinoma (HCC) is a devastating global health problem as the fifth most common malignancy and second greatest cause of cancer mortality worldwide (Degasperi and Colombo, 2016). HCC is often and increasingly caused by nonalcoholic fatty liver disease (NAFLD) (Dyson et al., 2014; Estes et al., 2018; Margini and Dufour, 2016; Younossi et al., 2015). Additionally, the two primary risk factors for NAFLD and NAFLD-mediated HCC are obesity and diabetes. (Margini and Dufour, 2016). Although the mechanisms underlying NAFLD-mediated HCC are not fully understood, factors arising from the deranged metabolism induced by chronic overnutrition are agreed to be causal factors. These include aberrant insulin and insulin growth factor 1 (IGF-1) signaling, chronic inflammation, and increased oxidative damage (Kutlu et al., 2018). As the obesity, diabetes, and NAFLD epidemics grow, new therapies are urgently needed to curb consequently increasing NAFLD-mediated HCC incidence (Baffy et al., 2012).

The recently identified mitochondrial pyruvate carrier (MPC) has emerged as a potential diabetes and NAFLD therapeutic target (Bricker et al., 2012; Colca et al., 2018; Gray et al., 2015; Herzig et al., 2012; McCommis et al., 2015, 2017; Rauckhorst et al., 2017). The MPC resides in the inner mitochondrial membrane and transports pyruvate from the cytosol into the mitochondrial matrix (Bricker et al., 2012; Herzig et al., 2012). Thus, the MPC occupies a critical metabolic node by linking glycolysis with mitochondrial metabolism. We and others demonstrated that liver-specific MPC disruption in diabetic mice decreases hyperglycemia (Gray et al., 2015; McCommis et al., 2015). Subsequent studies showed that chemical and genetic MPC disruption attenuates NAFLD by decreasing tricarboxylic acid (TCA) cycle flux and metabolite pool sizes, inflammation, and fibrosis (McCommis et al., 2017; Rauckhorst et al., 2017). Thus, MPC disruption could be expected to prevent HCC.

Conversely, hepatocyte MPC disruption decreases gluconeogenesis, thereby phenocopying a defining and consistent HCC feature (Facciorusso et al., 2016; Hirata et al., 2016; Khan et al., 2015; Likhitrattanapisal et al., 2016; Ma et al., 2013; Shang et al., 2016). Moreover, in several cancers MPC deficiency promotes the Warburg effect, sternness, and proliferation (Flores et al., 2017; Gray et al., 2015; Li et al., 2017a, 2017b; McCommis et al., 2015; Sandoval et al., 2017; Schell et al., 2017). Thus, a critical question is whether the anti-NAFLD or pro-HCC metabolism effects of MPC disruption dominate to decrease or increase HCC development. Furthermore, we considered that parsing this pro- and anti-cancer tension could isolate metabolic variables fundamentally underlying cancer development. Because chemical MPC inhibition is now being tested in clinical trials to treat NAFLD, this problem is also timely and clinically important (Colca et al., 2018). Therefore, we aimed to understand the contribution of the MPC to HCC development.

Here, we implemented the well-established diethylnitrosamine (DEN) plus carbon tetrachloride (CCl_4_) chronic oxidative stress mouse model of HCC development, which recapitulates human HCC genetic heterogeneity (Chappell et al., 2016; Marrone et al., 2016; Pound and McGuire, 1978; Uehara et al., 2014). Remarkably, compared to wild-type (WT) mice, liver-specific MPC knockout (MPC LivKO) mice developed two-thirds fewer liver tumors with increased tumor apoptosis. Using a combination of unbiased transcriptomic profiling, biochemical and cell-based assays, and stable-isotope metabolomic tracing, we discovered that MPC disruption reroutes glutamine away from glutathione synthesis into the TCA cycle as a mechanism for impaired hepatocellular tumorigenesis. Elevated mitochondrial glutamine anaplerosis is recognized as an important cancer mechanism for sustaining TCA cycle flux (Altman et al., 2016; Vander Heiden and DeBerardinis, 2017; Zhang et al., 2017). We show here, surprisingly, that mitochondrial glutamine utilization can be anticancer by competitively limiting glutathione synthesis, a key component of tumor initiation, promotion, and progression (Bansal and Simon, 2018; Harris et al., 2015; Huang et al., 2013; Lam et al., 2017).

## RESULTS

### *MPC1* and *MPC2* Are Highly Expressed in Human HCC

**Figure 1.**
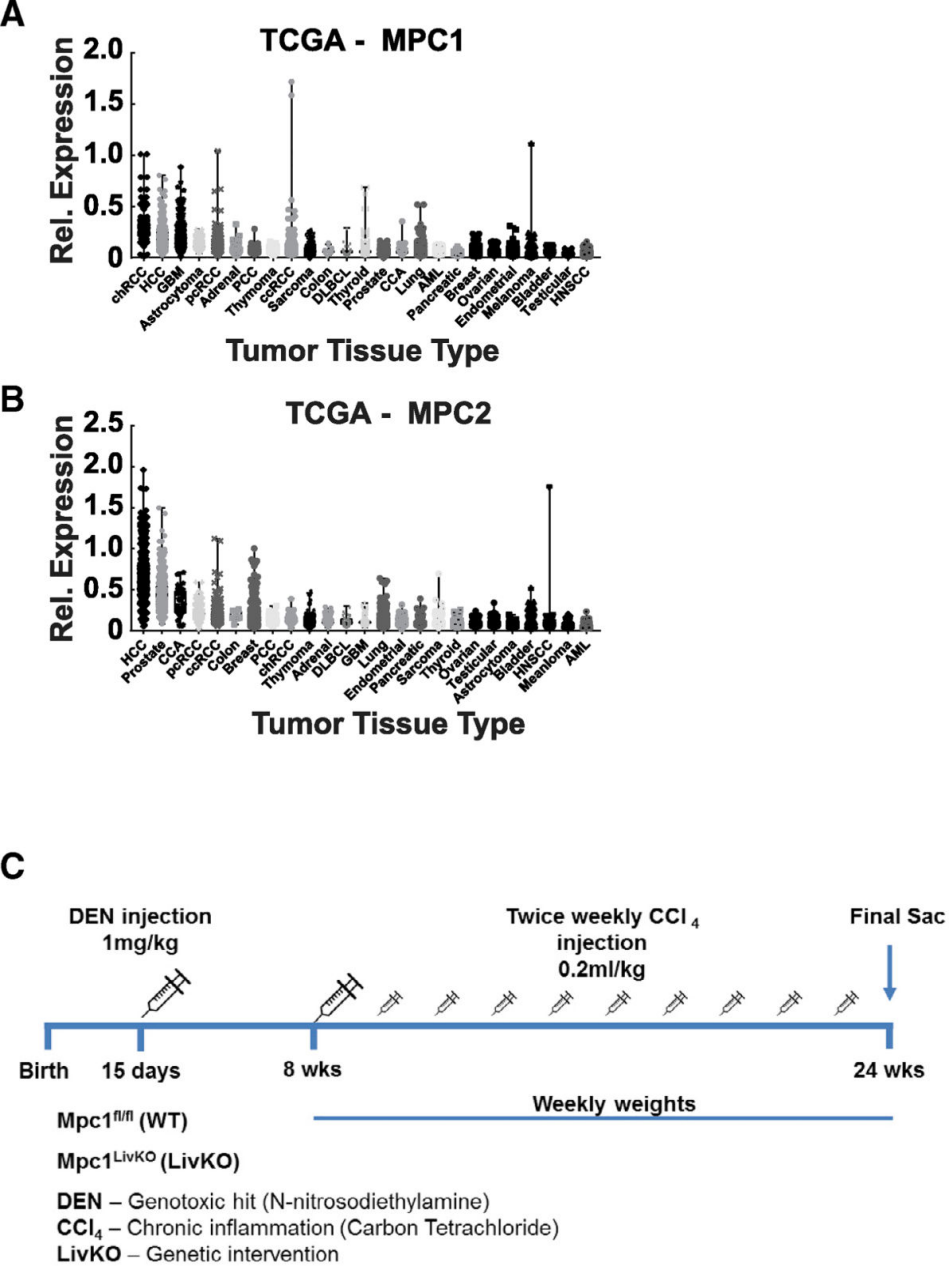
*MPC1* and *MPC2* Expression in the Tissue Cancer Genome Atlas (A and B) Expression of *MPC1* (A) and *MPC2* (B) in different cancer tissue types, ordered from highest to lowest median expression. Data acquired by cBioPortal (Cerami et al., 2012). (C) DEN plus CCl_4_ hepatocarcinogenesis model. WT and MPC LivKO mice were injected with DEN (1 mg/kg) on postnatal day 15. This was followed by twice-weekly (0.2 mL/kg) CCl_4_ injections with body weight measurements starting at 8 weeks of age. At 24 weeks of age, after 16 weeks of CCl_4_ injections, mice were euthanized.

The MPC is a heterodimer comprising two obligate protein subunits, namely, MPC1 and MPC2. Loss of either destabilizes the other and eliminates the MPC complex (Bricker et al., 2012; Herzig et al., 2012; Oonthonpan et al., 2019; Tavoulari et al., 2019). To understand the potential importance of MPC function in human HCC, we analyzed *MPC1* and *MPC2* expression in The Cancer Genome Database (TCGA), a comprehensive multidimensional map of key genomic changes in 33 types of cancer (Cerami et al., 2012). Within the TCGA, HCC was the second highest median *MPC1*-expressing and the highest median *MPC2*-expressing tumor type (Figures 1A and 1B). Retention and expression of MPC genes is striking because HCC universally downregulates the MPC-mediated process of gluconeogenesis (Gray et al., 2015; Ma et al., 2013; McCommis et al., 2015; Shang et al., 2016). Thus, the retained expression of *MPC1* and *MPC2* in TCGA HCCs suggested the MPC may have a yet-to-be-discovered pro-tumorigenic function.

### Liver-Specific MPC Disruption Decreases Hepatocellular Tumorigenesis

We previously observed that MPC LivKO mice are resistant to high-fat-diet-induced hyperglycemia, liver inflammation, and liver fibrosis (Gray et al., 2015; Rauckhorst et al., 2017). Here, we aimed to test whether this protective effect would extend to HCC prevention. We induced HCC in WT *(Mpc1*^*fl/fl*^*)* and MPC LivKO *(Mpc1*^*fl/fl*^*+Alb*^*Cre*^*)* by using a low-dose version of the well-established N-nitrosodiethylamine (DEN) plus carbon tetrachloride (CCl_4_) model (Uehara et al., 2014). DEN randomly mutates the genome, and CCl_4_ produces hepatic oxidative stress and inflammation, accelerating HCC development. In contrast to oncogene-induced HCC, the DEN/CCl_4_ model recapitulates the genetic heterogeneity of human HCC (Chappell et al., 2016; Marrone et al., 2016).

**Figure 2.**
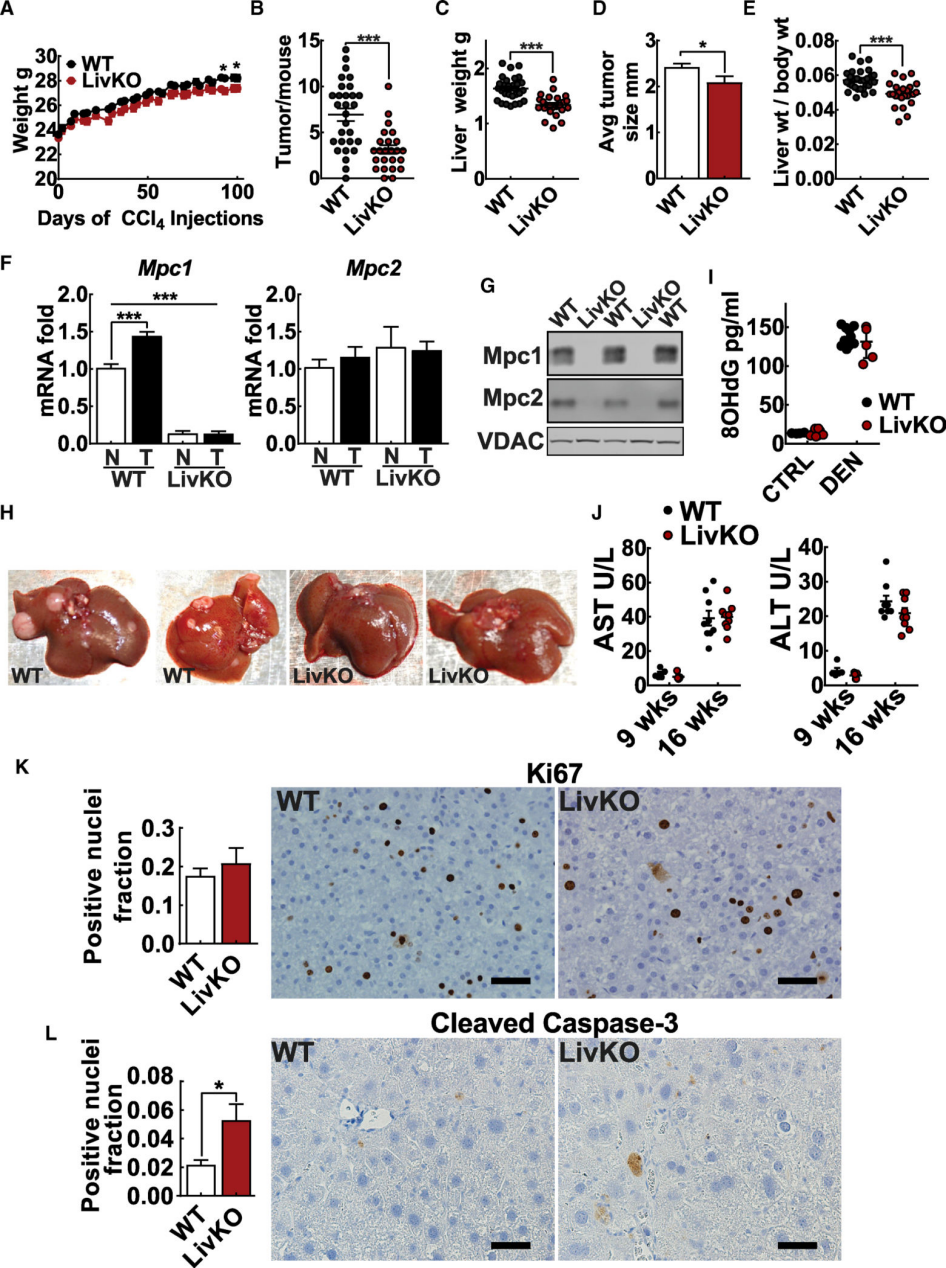
Hepatic Mpc1 Deletion Decreases Hepatocellular Carcinoma Development (A) Body weight of WT and MPC LivKO mice over course of CCl_4_ treatment; n = 24–29 biological replicates. (B) Liver tumor burden at euthanasia, determined by grossly dissecting each liver lobe and examining for and counting visible tumors; please see STAR Methods section for additional detail; n = 24–29 biological replicates C) Liver mass at euthanasia, determined by removing and weighing whole livers; n = 24–29 biological replicates. (D) Average tumor size, measured by calipers; n = 152 WT and 54 MPC LivKO tumors measured. (E) Calculated liver-to-body weight ratio for WT and MPC LivKO mice; n = 24–29 biological replicates. (F) *Mpc1* and *Mpc2* gene expression by qPCR in paired normal-adjacent non-tumor tissue (NT) and tumor tissue (T) in WT and MPC LivKO samples; n = 4 biological replicates per condition per genotype analyzed by one-way ANOVA. (G) Representative western blot of Mpc1 and Mpc2 expression in normal-adjacent non-tumor liver tissue of WT and MPC LivKO mice. VDAC loading control. (H) Representative liver images. (I) 8-OHdG ELISA on day 15 saline-injected (CTRL) or DEN-injected (DEN) pups; n = 4–11 biological replicates. (J) AST and ALT measured in plasma taken from WT and MPC LivKO mice after 9 weeks of CCl_4_ injections (n = 5, biological replicates) and prior to euthanasia; n = 10 biological replicates. (K) Ki67 staining of WT and MPC LivKO tumors; scale bar, 50 μm; n = 5–6 biological replicates. (L) Cleaved caspase-3 staining of WT and MPC LivKO tumors; scale bar, 50 μm; n = 5–6 biological replicates. Data are presented as mean ± SEM, compared by t test unless otherwise noted (*p < 0.05, **p < 0.01, ***p < 0.001). See also Figures S1, S2, and S3.

WT and MPC LivKO mice were injected with DEN (1 mg/kg) on postnatal day 15. This was followed by twice-weekly CCl_4_ injections (0.2 mL/kg), with body weight measurements starting at 8 weeks of age (Figure 1C). At 24 weeks of age, after 16 weeks of CCl_4_ injections, mice were euthanized and gross tumor burden was visually determined by two individuals blinded to liver genotype. Data from two separate cohorts generated 1 year apart are presented combined. Results from individual cohorts are shown in Figure S1. Animal weights did not differ throughout the study (Figure 2A). Compared to WT mice, MPC LivKO mice had significantly fewer tumors per liver, smaller average tumor size, smaller liver weights, and a smaller liver-to-body weight ratio (Figures 2B–2E). qPCR analysis of normal adjacent and paired tumor tissue confirmed *Mpc1* expression loss in MPC LivKO mice. Conversely, WT tumors compared to normal-adjacent tissue significantly increased *Mpc1* expression (Figure 2F). Western blots confirmed loss of both Mpc1 and Mpc2 protein in MPC LivKO livers (Gray et al., 2015; McCommis et al., 2015) (Figures 2G and S2A). These results indicate that MPC LivKO livers are less susceptible to chronic metabolic-injury-induced HCC. Representative images show decreased MPC LivKO tumor numbers (Figure 2H).

We considered whether genotypic differences in DEN-induced DNA damage could explain decreased MPC LivKO liver tumorigenesis. However, analyzing livers of 15-day-old mice 24 h after DEN injection for the DNA damage marker 8-OHdG revealed a 10-fold DNA damage increase compared to vehicle controls but no differences between genotypes (Figure 2I). Genotyping and western blots of these developing livers showed Cre to be present in MPC LivKO livers but that Mpc1 protein loss was not yet complete (Figures S2B and S2C). Furthermore, dihydroethidium (DHE) fluorescence of fresh-frozen liver slices from normal chow-fed, untreated mice was not different between genotypes, consistent with no difference in basal reactive oxygen species production (Figure S3). Measuring the plasma liver damage markers aspartate aminotransferase (AST) and alanine aminotransferase (ALT) after 9 weeks of CCl_4_ treatment and at time of sacrifice (16 weeks of CCl_4_ treatment) also demonstrated no difference between genotypes (Figure 2J). These measurements suggest that attenuated MPC LivKO mouse HCC development cannot be explained by diminished CCl_4_ liver injury susceptibility.

### MPC LivKO Tumors Exhibit Increased Apoptosis

Because MPC LivKO tumors were smaller than WT tumors, we questioned if they had decreased proliferation or increased apoptosis. We performed immunohistochemistry on liver tumor sections for a cell proliferation marker, Ki67, and an apoptosis marker, cleaved-caspase 3. WT and MPC LivKO livers showed no difference in the Ki67-positive nuclei fraction, consistent with no proliferation rate difference (Figure 2K). Compared to WT, MPC LivKO livers showed significantly increased cleaved-caspase-3-positive nuclei, indicating a higher apoptosis rate (Figure 2L).

### RNA Sequencing Reveals Genotype Divergent Changes in Glutathione Metabolism Genes

**Figure 3.**
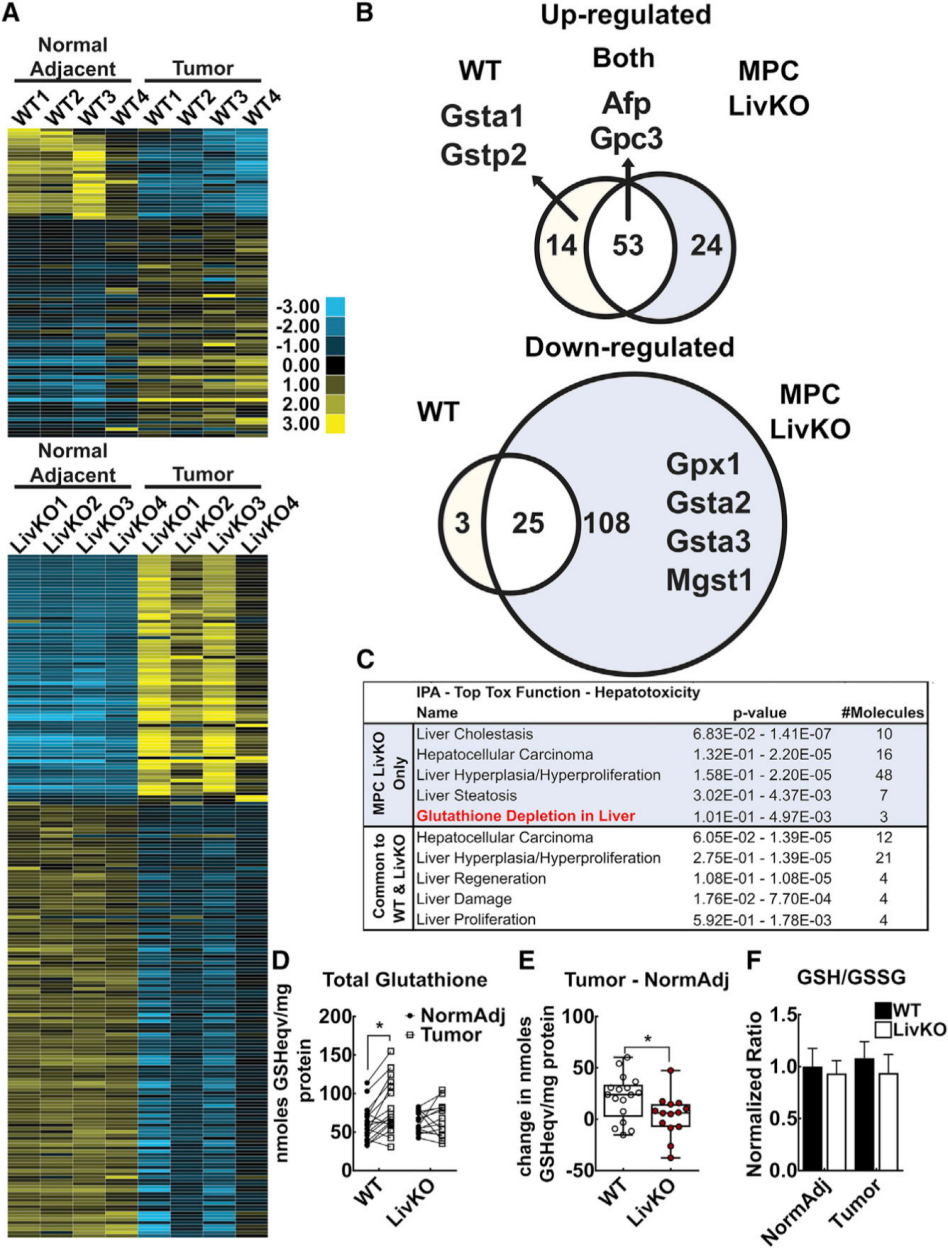
Transcriptomic and Enzymatic Measurements Identify Glutathione Stress in MPC LivKO Tumors (A) Unsupervised clustering of significantly changed gene expression in tumors compared to paired normal adjacent tissue; n = 4 biological replicates. (B) Venn diagram of gene expression clusters with glutathione-related and HCC marker genes. (C) Significantly changed pathways based on RNA-sequencing-derived gene clusters identified by Ingenuity Pathway Analysis (IPA). The IPA pathways are identified in different groups because different genes in the same IPA pathway are changed between groups. (D) Total glutathione (GSH + GSSG) levels measured in paired normal-adjacent and tumor tissue taken from WT and MPC LivKO mice at euthanasia; n = 15–17 biological replicates. (E) The data in (D) presented as mean change in total glutathione levels; n = 15–17 biological replicates. (F) Reduced glutathione (GSH) to oxidized glutathione (GSSG) ratios in WT and MPC LivKO normal-adjacent and tumor tissue. Ratios are normalized to WT NormAdj levels. n = 15–17 biological replicates. For statistical analysis, data were analyzed by two-way ANOVA. Data are presented as mean ± SEM, compared by t test performed unless otherwise noted (*p < 0.05, **p < 0.01, ***p < 0.001).

To understand how MPC loss resulted in markedly fewer and slightly smaller tumors with a higher apoptosis rate, we sought to identify signatures of genetic selection. We performed RNA sequencing to compare WT and MPC LivKO tumor and paired normal-adjacent tissue transcript levels. Unsupervised clustering analysis identified six gene expression change groups: (1) increased in both WT and LivKO tumors; (2) increased in WT tumors; (3) increased in MPC LivKO tumors; (4) decreased in both WT and LivKO tumors; (5) decreased in WT tumors; and (6) decreased in MPC LivKO tumors (Figures 3A and 3B; Table S1). The two clinically utilized human HCC markers Alpha-fetoprotein *(Afp)* and Glypican-3 *(Gpc3)* were upregulated in both WT and MPC LivKO tumors, providing evidence of similarity to human HCC (Masuzaki et al., 2012). Indeed, Ingenuity Pathway Analysis (IPA) identified the HCC pathway as common to both MPC LivKO and WT tumors (Figure 3C).

Interestingly, RNA sequencing data revealed genotype-divergent expression of glutathione-consuming genes. In the cluster of 14 genes upregulated in WT tumors, two, *Gsta1* and *Gstp2,* are both glutathione-S-transferases (GSTs). GSTs detoxify peroxidized lipid aldehyde derivatives and electrophilic xenobiotics by glutathione conjugation, decreasing toxicity and facilitating elimination. Conversely, three GSTs, *Gsta2, Gsta3,* and *Mgst1* were in the cluster of 108 genes downregulated in MPC LivKO tumors. The same cluster contained glutathione peroxidase *(Gpx1),* another glutathione consuming enzyme. Thus, WT tumors increased but MPC LivKO tumors decreased expression of glutathione metabolizing genes. Strikingly, IPA identified the pathway “Glutathione Depletion in Liver” as specific to MPC LivKO tumor samples (Figure 3C). These RNA sequencing and IPA results led us to consider whether glutathione depletion due to MPC disruption could be a mechanism for impaired HCC development.

### The MPC Mediates Increased Tumor Glutathione Content

Glutathione, an anti-oxidant tripeptide, is critical for tumor initiation, and glutathione levels are elevated in HCC and correlate with cell growth rate (Harris et al., 2015; Huang et al., 2013; Lam et al., 2017). Therefore, we measured glutathione content in tumor and normal adjacent tissue. WT normal tissue and MPC LivKO normal tissue samples did not have different mean total glutathione levels. However, compared to adjacent normal tissue, WT tumors, but not LivKO tumors, increased total glutathione (Figure 3D). Similarly, when measured as net change in glutathione from each normal-adjacent sample to its paired tumor sample, WT tumors increased total glutathione levels significantly more than MPC LivKO tumors (Figure 3E). The reduced glutathione (GSH) to oxidized glutathione (GSSG) ratio was not different between tumor and adjacent normal tissue and was unaffected by genotype, suggesting unchanged overall thiol redox balance under the basal conditions of tissue harvest (Figure 3F). The inability of MPC LivKO tumors to increase total glutathione levels suggest decreased synthesis, increased consumption, or both.

### MPC Disruption in Cultured Hepatoma Cells Decreases Glutathione Content and Is Synthetically Lethal with Glutathione Depletion

**Figure 4.**
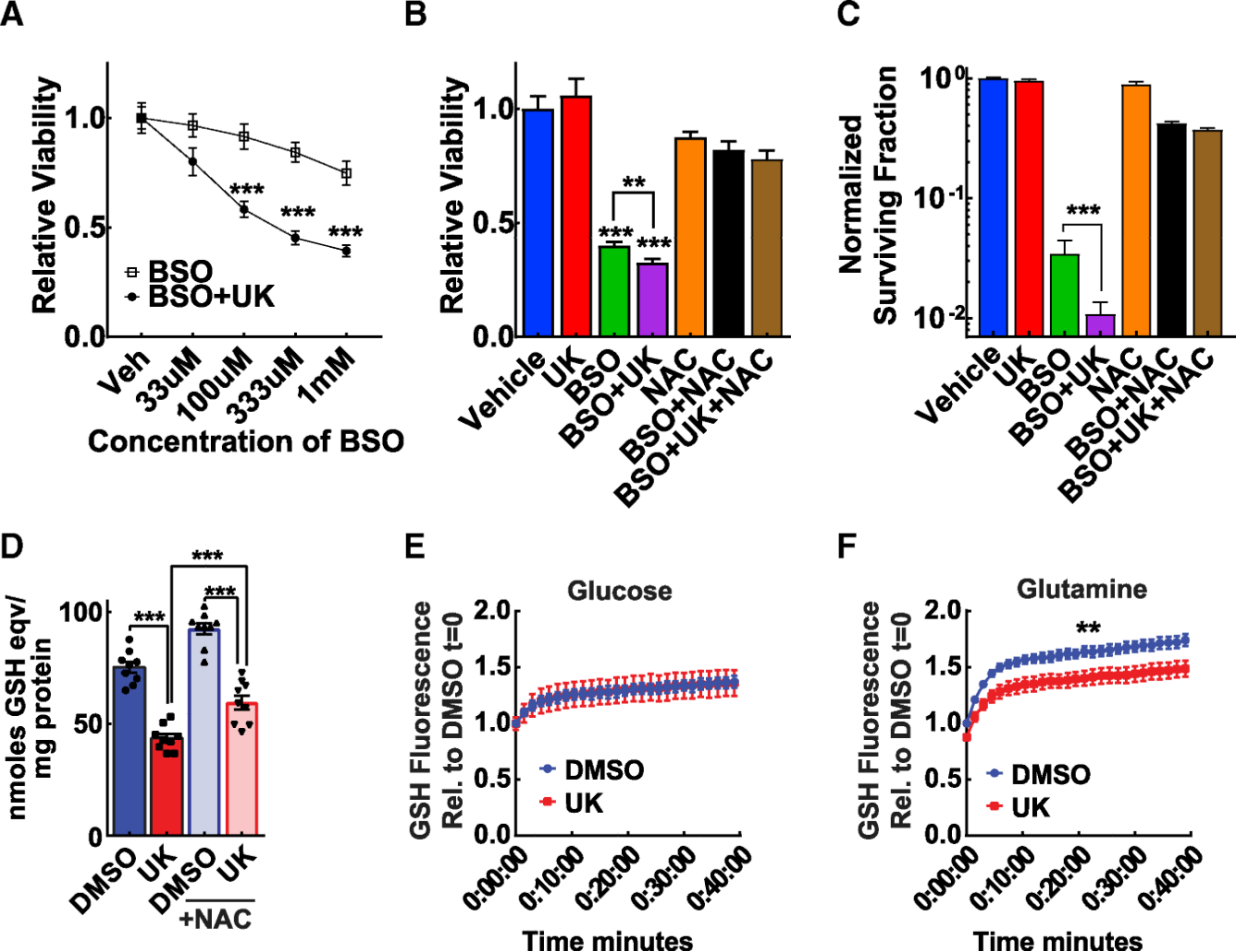
MPC Disruption Decreases Viability and Glutathione Content in Hepa1–6 Cells and Primary Hepatocytes (A) Hepa1–6 cells treated for 48 h with 5 μM UK5099 (UK) or 5 μM UK5099 (UK) + varied concentrations of BSO. Viability measured by resazurin assay; n = 16–18 replicate wells. (B) Hepa1–6 cells treated for 48 h with DMSO (Vehicle), 5 μM UK5099 (UK), 1 mM buthionine sulfoximine (BSO), 1 mM BSO + 5 μM UK5099, 5 mM N-acetyl cysteine (NAC), 1 mM BSO + 5 mM NAC, or 1 mM BSO + 5 μM UK5099 + 5 mM NAC. Viability measured by resazurin assay; n = 9–17 replicate wells. (C) Clonogenic survival of Hepa1–6 cells treated with 5 μM UK5099, 1 mM BSO, 1 mM BSO + 5 μM UK5099, 10 mM NAC, 1 mM BSO + 10 mM NAC, or 1 mM BSO + 5 μM UK5099 + 10 mM NAC for 72 h. For statistical analysis, data were log-transformed before performing a one-way ANOVA; n = 8–12 replicates. (D) Total glutathione (GSH) levels in Hepa1–6 cells treated with either DMSO or 5 μM UK5099 in the presence of absence of NAC for 48 h; n = 9 replicate wells. (E and F) Monochlorobimane (mBCl) GSH assay in primary hepatocytes, 5 mM glucose (E), or 5 Mm glucose supplemented with 5 mM glutamine (F), treated with DMSO or 5 μM UK5099; n = 4–5 biological replicates. Data are presented as mean ± SEM, compared by t test unless otherwise noted (*p < 0.05, **p < 0.01, ***p < 0.001). See also Figure S4.

After observing the impaired ability of MPC LivKO tumors to increase glutathione levels, we sought to investigate, first, if glutathione loss decreases hepatoma viability and, second, if acutely disrupting MPC activity decreased glutathione levels. We first tested how the specific MPC inhibitor UK5099 affected Hepa1–6 mouse hepatoma cell viability. To induce glutathione stress, proliferating Hepa1–6 cells were treated with the glutathione-depleting agent buthionine sulfoximine (BSO) and either UK5099 or vehicle for 48 h. Control experiments confirmed that 48-h BSO treatment depleted Hepa1–6 glutathione (Figure S4A). BSO treatment dose-dependently decreased cell viability measured by resazurin reduction, and this effect was amplified by combined UK5099 treatment (Figure 4A). This demonstrated a synthetic effect of MPC inhibition and glutathione depletion. Notably, UK5099 treatment did not affect cell viability compared to vehicle (Figure 4B). We next questioned whether viability could be rescued by the thiol antioxidant and partial glutathione precursor N-acetylcysteine (NAC). Indeed, BSO + UK5099-treated Hepa1–6 cell viability was partially rescued by NAC supplementation when measured by resazurin reduction (Figure 4B) or crystal violet staining (Figure S4B).

To test the effect of glutathione depletion and MPC disruption on clonogenic potential, we treated Hepa1–6 cells with BSO, UK5099, and NAC combinations for 72 h and then plated cells for colony-forming assays. BSO + UK5099-treated cells generated about two-thirds fewer colonies than BSO-treated cells (Figure 4C). This demonstrates that glutathione depletion and MPC disruption synthetic lethality extend to clonogenic potential, the preferred culture model for tumorigenic potential. We then measured total glutathione levels in Hepa1–6 cells treated with either DMSO or UK5099 for 48 h. MPC inhibition significantly decreased total glutathione, recapitulating the decreased glutathione observed in MPC LivKO versus WT tumors (Figure 4D).

Given that MPC inhibition decreased glutathione levels in Hepa1–6 hepatoma cells, we next tested how MPC inhibition affects glutathione synthesis in mouse primary hepatocytes, the site of *in vivo* HCC initiation. To enable real-time measurements of cellular GSH levels, we utilized the small molecule monochlorobimane (mBCl), which is rapidly conjugated to GST, forming a fluorescent product. Mouse primary hepatocytes were supplemented with 5 mM glucose and treated with either DMSO or UK5099. Importantly, because mBCl can react with free thiols, we note that experimental media contained the oxidized disulfide dimer of cysteine, cystine, but not free cysteine. No difference in GSH-dependent fluorescence was observed between groups (Figure 4E). Glutamine is a key glutamate source for glutathione synthesis. Because glutamine can directly supply glutamate for glutathione synthesis, we repeated the experiment with 5 mM glutamine in addition to 5 mM glucose. DMSO-treated hepatocytes produced significantly more GSH-dependent fluorescence than MPC-inhibited primary hepatocytes (Figure 4F). These results demonstrate that acute chemical MPC inhibition decreases glutamine-dependent glutathione content.

### Stable Isotope Tracing Reveals MPC Disruption Reroutes Glutamine Carbon into the TCA Cycle and away from Glutathione

We observed MPC disruption decrease glutathione levels in MPC LivKO tumors, Hepa1–6 cells, and glutathione-stressed primary hepatocytes. We considered that glutamine is utilized as both a TCA cycle fuel and precursor for glutathione synthesis and, thus, could competitively link these fundamental cellular processes. We aimed to test the hypothesis that MPC-disrupted hepatocytes re-route glutamine toward the TCA cycle, away from glutathione synthesis. We performed experiments *in vivo* with whole liver and *ex vivo* with primary hepatocytes to survey glutamine carbon partitioning between the TCA cycle and glutathione. To most acutely capture effects of *in vivo* MPC disruption, we utilized *Mpc1*^*fl/fl*^ mice that were treated with adeno-associated virus (AAV)-Cre (MPC LivKO) or AAV-empty vector (WT), expressed from the TBG promoter, for potent, hepatocyte-selective recombination (Gray et al., 2015; Rauckhorst et al., 2017; Yan et al., 2012). We intravenously administered universally (U)^13^C-labeled glutamine tracers to measure glutamine flux into metabolites. Here, each of the five glutamine carbon atoms is the stable, ^13^C isotope, resulting in a 5-Dalton mass increase (M+5). (U)^13^C-glutamine (400 mg per kg body weight) was administered to WT and MPC LivKO mice by two retro-orbital injections 15 min apart. Livers were harvested and immediately freeze-clamped at 30 min after the first injection.

**Figure 5.**
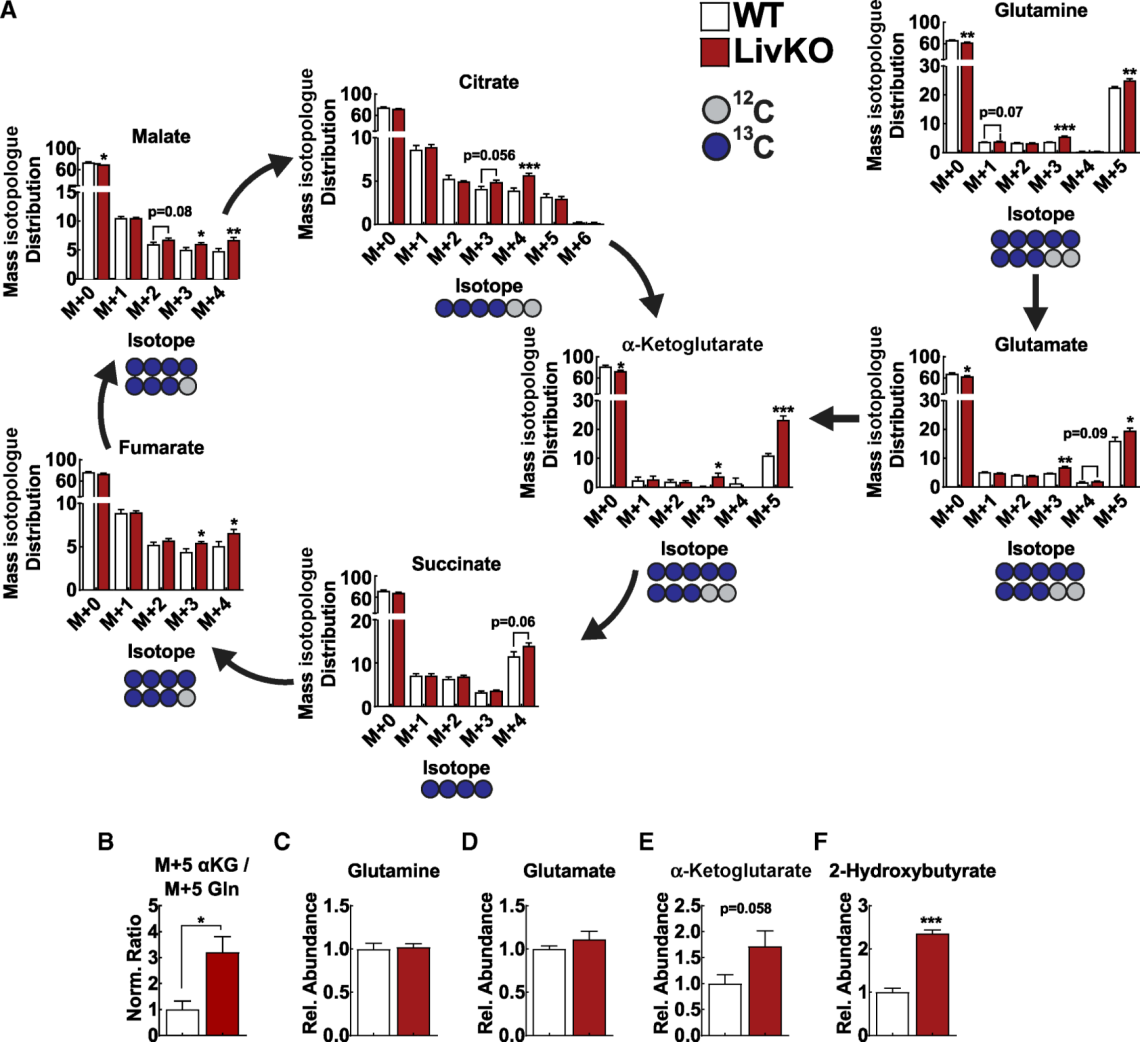
*In Vivo* (U)^13^C Glutamine Tracing Demonstrates Increased TCA Cycle Glutamine Utilization in MPC LivKO Livers (A) Traced isotopologue distribution in liver tissue after WT (white) and MPC LivKO (red) mice were intravenously injected with 400 mg/kg (U)^13^C glutamine. M+n corresponds to the number, n, of ^13^C carbons being incorporated into the metabolite; n = 6–7 biological replicates. (B) Mean normalized ratio of the M+5 α-ketoglutarate signal to the M+5 glutamine signal for WT and MPC LivKO; n = 6–7 biological replicates. (C–F) Relative abundance of glutamine (C), glutamate (D), α-ketoglutarate (E), and 2-hydroxybutyrate (F) in WT and MPC LivKO livers; n = 6–7 biological replicates. Data are presented as mean ± SEM, compared by t test performed unless otherwise noted (*p < 0.05, **p <0.01, ***p < 0.001). See also Figure S5 and Tables S2 and S3.

Hepatic extracts were analyzed by gas chromatographymass spectroscopy (GC-MS) metabolomic profiling and stable isotope tracing. Principal-component analysis (PCA) confirmed the WT and MPC LivKO liver metabolomes to be systematically different (Figure S5A). Mass isotopologue analysis revealed the M+5 glutamine, M+5 glutamate, M+5 α-ketoglutarate, M+4 succinate, M+4 fumarate, M+4 malate, and M+4 citrate isotopologue fractions to be greater in MPC LivKO mice (Figure 5A; Table S2). This demonstrates greater enrichment of M+5 glutamine into the TCA cycle of MPC LivKO livers. As an additional measure of mitochondrial glutamine utilization, we examined the ratio of M+5 α-ketoglutarate to M+5 glutamine. MPC LivKO livers manifested a nearly 3-fold increase in this ratio, consistent with a 3-fold increase in TCA cycle glutamine entry (Figure 5B).

To understand how glutamine and α-ketoglutarate total concentration differences could contribute to ^13^C-enrichment differences, we measured metabolite pool sizes (Table S3). Metabolite profiling revealed WT and MPC LivKO liver glutamine and glutamate levels to be identical (Figures 5C and 5D). Notably, MPC LivKO liver α-ketoglutarate trended greater (p = 0.058), whereas other TCA cycle intermediates were decreased or not different (Figures 5E and S5B). The unchanged glutamine but increased α-ketoglutarate pool size corroborate ratiometric comparisons of M+5 glutamine and M+5 α-ketoglutarate isotopologue fractions. This is consistent with increased glutamine channeling into the TCA cycle through α-ketoglutarate to compensate for lost MPC LivKO mitochondrial pyruvate uptake. Notably, total metabolite profiling also revealed MPC LivKO livers to have more than two-fold greater 2-hydroxybutyrate. 2-hydroxybutyrate is a specific marker of glutathione stress and production by the transsulfuration pathway (Lord and Bralley, 2008). Thus, increased 2-hydroxybutyrate in MPC LivKO livers is consistent with adaptive glutathione synthesis (Figure 5F).

**Figure 6.**
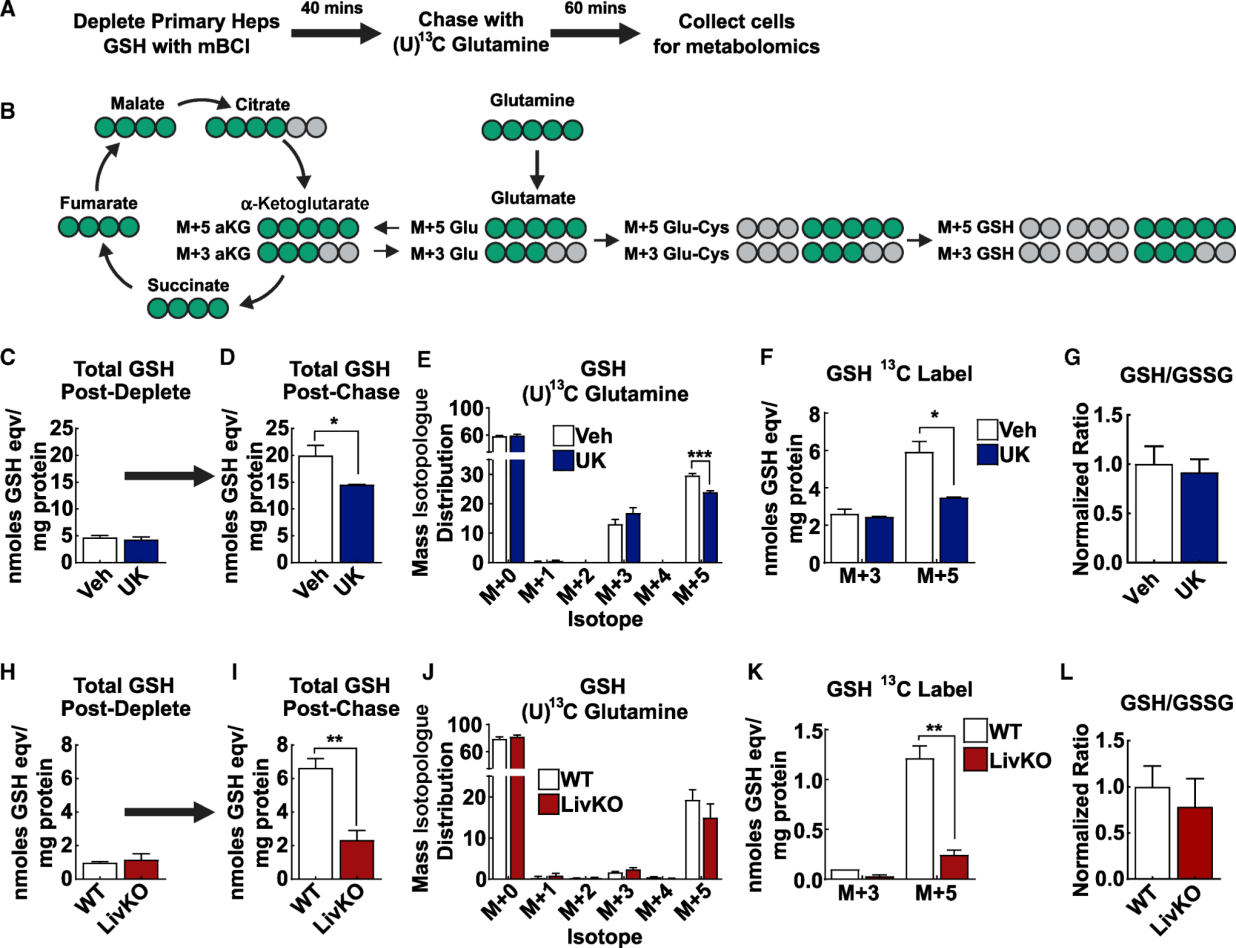
*Ex Vivo* (U)^13^C Glutamine Tracing Demonstrates MPC Disruption Impairs Hepatocyte Glutathione Synthesis (A) Schematic illustration of the time course for glutathione depletion and (U)^13^C glutamine tracing in primary hepatocyte experiments. (C–G) Primary hepatocytes were isolated from 12-week-old WT mice. (H–L) Primary hepatocytes isolated from 17- to 20-week-old WT and MPC LivKO mice. (B) Schematic demonstrating metabolic path U-^13^C glutamine incorporation into glutathione or the TCA cycle. Green denotes ^13^C label, and gray denotes ^12^C non-label. (C) Total glutathione determined enzymatically in primary hepatocytes following glutathione depletion induced by 40 min of treatment with 50 μM monochlorobimane; n = 3 biological replicates. (D) Total glutathione determined after 1 h of (U)^13^C glutamine chase following glutathione depletion as in (A). During the chase period, primary hepatocytes were incubated with 5 mM (U)^13^C glutamine, 5 mM glucose, and DMSO (Veh) or 5 μM UK5099 (UK); n = 3 biological replicates. (E) Glutathione mass isotopologue distribution after chase period described in (A); n = 3. (F) Concentration of M+3 and M+5 glutathione calculated by multiplying total glutathione shown in (C) with the isotopologue fractional enrichment shown in (D); n = 3 biological replicates. (G) GSH to GSSG ratios in DMSO (Veh)- and UK5099 (UK)-treated hepatocytes. Ratios are calculated from metabolite mass spectrometry signal intensity and normalized to vehicle. (H) Total glutathione determined enzymatically in WT and MPC LivKO primary hepatocytes following glutathione depletion as in (A); n = 4 biological replicates. (I) Total glutathione determined in WT and MPC LivKO primary hepatocytes after 1 h of (U)^13^C glutamine chase following glutathione depletion as in (A). Primary hepatocytes were incubated with 5 mM (U)^13^C glutamine, 5 mM glucose; n = 3 biological replicates. (J) Glutathione mass isotopologue distribution after chase period described in (A); n = 4. (K) Concentration of M+3 and M+5 glutathione calculated by multiplying total glutathione shown in (I) with the isotopologue fractional enrichment shown in (J); n = 3 biological replicates. (L) GSH to GSSG ratios in WT and MPC LivKO hepatocytes. Ratios are calculated from metabolite mass spectrometry signal intensity and normalized to WT; n = 4 biological replicates. Data are presented as mean ± SEM, compared by t test performed unless otherwise noted (*p < 0.05, **p < 0.01, ***p < 0.001). See also Figure S6.

To mechanistically test for glutathione synthesis differences arising from MPC disruption, we moved from an *in vivo* glutamine tracer model to a more malleable primary hepatocyte tracer model. Importantly, a goal of this experiment was to reasonably recapitulate how MPC disruption affects glutamine metabolism during the high glutathione stress and genetic selection during hepatocyte transformation, which cannot be measured in established tumors. To examine glutamine flux into glutathione synthesis during acute glutathione stress, primary hepatocyte glutathione was depleted by mBCl chemical conjugation. This was followed by incubating primary hepatocytes in media containing (U)^13^C-labeled glutamine, with either vehicle or UK5099 treatment (Figure 6A). (U)^13^C-labeled glutamine can generate either M+3 or M+5 glutathione, depending on whether it is first oxidatively routed through the TCA cycle before incorporation into glutathione (Figure 6B). Primary hepatocyte total glutathione levels were similarly depleted by mBCl treatment (Figure 6C). Compared to vehicle, hepatocytes treated with the MPC inhibitor UK5099 had significantly less total glutathione (Figure 6D).

Next, liquid chromatography-mass spectrometry (LC-MS), which, in contrast to GC-MS, enables detection of intact glutathione, mass isotopologue analysis revealed that the M+5 glutathione fraction of total glutathione was significantly less in UK5099-treated hepatocytes (Figure 6E). Thus, MPC-inhibited hepatocytes had decreased direct flux of (U)^13^C-glutamine into glutathione synthesis. The concentration of newly synthesized M+5 glutathione was then calculated by multiplying the M+5 isotopologue fraction by total glutathione concentration. UK5099-treated hepatocytes had a significantly decreased M+5 glutathione isotopologue total concentration (Figure 6F). Importantly, MPC inhibition did not change the GSH to GSSG ratio, which is consistent with impairment of glutathione synthesis versus modulation of thiol redox state (Figure 6G).

We repeated this experiment with WT and MPC LivKO primary hepatocytes. mBCl treatment similarly depleted WT and MPC LivKO hepatocyte total glutathione levels (Figure 6H). Compared to WT, MPC LivKO hepatocytes re-synthesized less total glutathione and had a decreased M+5 glutathione total concentration, demonstrating impaired glutathione synthesis (Figures 6J and 6K). MPC deletion did not change the GSH to GSSG ratio (Figure 6L). To enable ratiometric comparison of M+5 glutathione enrichment to M+5 glutamine loading, we modified our mass spectrometry analytical workflow from that used for the immediately preceding experiments using UK5099 to disrupt MPC activity to also detect intact glutamine. As expected, compared to WT hepatocytes, MPC LivKO hepatocytes exhibited a significantly decreased M+5 glutathione-to-M+5 glutamine ratio, further demonstrating that MPC disruption decreases glutamine flux into glutathione (Figure S6). Together, these data demonstrate that MPC-disrupted hepatocytes increase glutaminolysis to maintain the TCA cycle, re-synthesize glutathione at a slower rate, and utilize less glutamine for glutathione synthesis.

## DISCUSSION

Type 2 diabetes and NAFLD are now the fastest growing HCC risk factors due to the availability of hepatitis B vaccines and hepatitis C treatments (Estes et al., 2018; Facciorusso et al., 2016; Ferlay et al., 2010; Kutlu et al., 2018; Margini and Dufour, 2016; Schütte et al., 2016). Thus, the changing etiology of HCC requires new prevention strategies. Disrupting liver MPC activity has recently been shown to attenuate diabetes and NAFLD (Gray et al., 2015; McCommis et al., 2015, 2017; Rauckhorst et al., 2017). This led us to question if MPC disruption could attenuate HCC development. However, given that MPC disruption induces a canonical, HCC-like metabolism by decreasing gluconeogenesis, an expected result was not necessarily clear (Gray et al., 2015; McCommis et al., 2015). We found that MPC disruption markedly decreased HCC development and discovered a previously unrecognized relationship among mitochondrial anaplerosis, glutamine metabolism, and tumorigenesis.

To understand the contribution of the MPC to HCC development, we utilized liver-specific MPC knockout (MPC LivKO) mice and a spontaneous, chronic metabolic-injury-driven HCC model. Strikingly, compared to WT mice, MPC LivKO mice developed two-thirds fewer tumors. Moreover, MPC LivKO tumors were smaller with increased apoptosis. In previous investigations, increased pyruvate anaplerosis in NAFLD increased TCA cycle flux, oxidative stress, and inflammation (Satapati et al., 2012, 2015). In two similar studies, liver MPC disruption prevented NAFLD-mediated TCA cycle metabolite pool expansion and decreased markers of inflammation and fibrosis (McCommis et al., 2017; Rauckhorst et al., 2017). Here, we demonstrate that MPC disruption markedly decreased chronic metabolic-injury-driven tumorigenesis. This result contributes to the growing body of literature illuminating how decreasing hepatic mitochondrial pyruvate uptake protects from progressive liver disease and demonstrates that decreasing gluconeogenesis is not necessarily oncogenic.

Our results reveal that metabolic competition for glutamine may be exploited to impair tumorigenesis. Through biochemical and stable isotope tracing assays, we found that loss of hepatocyte MPC activity limits glutathione synthesis. MPC disruption has previously been shown to increase mitochondrial glutamine utilization in cancer models, primary cortical neurons, and skeletal muscle and, conversely, to decrease glutamine utilization in incubated mouse retinas (Bader et al., 2018; Divakaruni et al., 2017; Grenell et al., 2019; Sharma et al., 2019; Vacanti et al., 2014; Yang et al., 2014). Our work here advances the understanding of cancer glutamine metabolism by demonstrating, first, that glutamine partitioning between TCA cycle oxidation and glutathione synthesis can be competitive and, second, that the MPC controls hepatocellular tumorigenesis, likely by modulating glutamine partitioning between these two fates.

Glutathione is critical for tumor initiation, a time of high reactive oxygen species (ROS), across multiple tumor types (Carteret al., 2016; Harris et al., 2015; Orrù et al., 2018). In numerous tumor types, glutathione levels positively correlate with resistance to treatment, invasiveness, and metastasis (Bansal and Simon, 2018; Hatem et al., 2017). Glutathione may also be consumed by GSTs. GSTs conjugate glutathione to xenobiotic and endogenous waste molecules, facilitating excretion. GSTs of the alpha class (GSTαs) are predominately expressed in the liver and comprise the majority of differentially expressed isoforms in our RNA sequencing dataset. The WT tumor GSTα upregulation and MPC LivKO tumor GSTα downregulation are consistent with glutathione scarcity limiting MPC LivKO HCC development and an adaptive glutathione sparing response. Hepatocytes require extraordinarily high glutathione synthesis rates for conjugating chemical waste during detoxification metabolism (Geenen et al., 2013; Pajares and Pérez-Sala, 2018). Thus, MPC disruption may exploit a metabolic vulnerability to which developing HCCs are inherently sensitive, leading to lethal glutathione depletion during the stress of HCC initiation and early progression.

Notably, here, hepatoma experiments differ from *in vivo* tumorigenesis experiments by two critical parameters. First, MPC inhibition in hepatomas was acute, and second, *in vivo* tumorigenesis selected for MPC LivKO HCCs more resistant to glutathione stress. In contrast to the high cell death observed in hepatoma clonogenic assays, the elevated but still minimal apoptosis observed in MPC LivKO versus WT tumors does not detect apoptosis at the critical threshold of tumor initiation, which was likely higher. Thus, the greater glutathione depletion observed in acutely MPC-inhibited hepatomas may better recapitulate focal glutathione depletion in pre-neoplastic lesions and mitotic catastrophe of transformed cells, before selection and progression to HCC.

Lastly, we note limitations of this work and potential future directions. Although the CCl_4_ tumorigenesis protocol used here is a more extreme oxidative stress than encountered by human livers during obesity and diabetes, we expect that the low-dose CCl_4_ model we utilized increases translational fidelity compared to higher dose CCl_4_ or single oncogene HCC models. To determine how well results here translate to obesity and NAFLD-mediated HCC, it will be important to test the effects of MPC disruption in chronic overnutrition models of HCC development. Furthermore, although the combination of *in vivo* and *ex vivo* culture approaches used here address how chronic MPC disruption changes hepatocellular tumorigenesis and how acute MPC disruption and glutathione depletion stress changes hepatoma survival, we did not test how acute MPC disruption affects HCC growth and survival nor how acute or chronic MPC disruption affects HCC invasiveness and metastatic potential. Thus, important future directions include determining how hepatocyte MPC disruption changes pre-neoplastic lesion glutathione metabolism and survival and how acute and chronic MPC disruption change clinically relevant advanced HCC outcomes.

In conclusion, we report that MPC disruption in hepatocytes decreased metabolic-injury-driven hepatocellular tumorigenesis. We demonstrate that adaptive mitochondrial glutamine utilization resulting from MPC disruption limits glutamine availability for glutathione synthesis. Taken together, our work reveals a previously unrecognized link between pyruvate metabolism and glutathione content and identifies the MPC as a plausible HCC prevention target. Our findings raise the possibility that combining MPC disruption and glutathione stress may be therapeutically useful in HCC and additional cancers.

## STAR★METHODS

### LEAD CONTACT AND MATERIALS AVAILABILITY

Further information and requests for resources and reagents should be directed to and will be fulfilled by the Lead Contact, Eric Taylor (eric-taylor@uiowa.edu). This study did not generate new unique reagents.

### EXPERIMENTAL MODEL AND SUBJECT DETAILS

#### Mouse Models

Animal work was performed in accordance with the University of Iowa Animal Care and Use Committee (IACUC). Mice were group housed up to 5 mice/cage and maintained on Harlan Scientific 2920i diet. Littermate paired mice were used in all experiments. Constitutive, hepatocyte-specific Mpc1 knockout and wild-type mice were generated as previously reported (Gray et al., 2015). Briefly, Mpc1^fl/fl^ mice were crossed with Mpc1^fl/fl^+Alb^Cre/+^ producing Mpc1^fl/fl^+Alb^Cre/+^ (LivKO) and Mpc1^fl/fl^ (WT) mice. Acute, hepatocyte-specific Mpc1 knockout (LivKO) and wild-type (WT) mice were generated by retro-orbitally injecting Mpc1^fl/fl^ mice with AAV8.TBG.PI.Cre.rBG (AAV-Cre, LivKO) or AAV8.TBG.PI.Null.bGH (AAV-EV, WT) using 1×10^11^ genome copies per mouse.

For the tumorigenesis studies: at 15 days of age mice were treated with 1 mg/kg N-nitrosodiethylamine (DEN) intraperitoneally. A 100 mg/ml stock solution of DEN was prepared in PBS and filtered. On injection day, 1 mg (10 μL of 100 mg/ml stock solution) DEN was added to 15 mL of filtered PBS to make a DEN injection solution of 0.067 μg/μl. Mice were then injected with 1 mg/kg. Depending on body weight, injection volume ranged from approximately 75 μL to 125 μl. A 40 μl/ml stock solution of carbon tetrachloride (CCl_4_) was prepared by mixing 400 μL CCl_4_ to 9.6 mL of filtered corn oil. Mice were injected with 0.2 ml/kg CCl_4_ twice weekly from age 8 to 24 weeks.

#### Cell Lines

Hepa1–6 cells were cultured in High Glucose DMEM supplemeted with 10% Fetal Bovine Serume, GlutaMAX, and 1% Penicillin-Streptomycin. Cells were cultured at 37°C with 20% oxygen and 5% carbon dioxide.

### METHOD DETAILS

#### Primary Hepatocyte Isolation

Mpc1^fl/fl^ mice treated with AAV-Cre (LivKO) or AAV-EV (WT) were used to harvest primary hepatocytes as described previously (Gray et al., 2015). Briefly, livers were first perfused with a buffered collagenase and trypsin inhibitor. Hepatocytes were liberated from the liver capsule. This initial cell suspension was centrifuged at 50 × *g* for 6 min, and the supernatant, enriched with Kupffer and other non-hepatocyte (non-parenchymal) cell types was discarded. The hepatocyte-enriched pellet was washed by gentally resuspending the cell pellet followed by centrifuging for 3 minutes at 50 × *g* to further remove debris, dead cells, and non-parenchymal cells. This washing step was repeated for a total of 2 washes. Hepatocytes were plated at a density of 166,666 cells/cm^2^ and allowed to attach for 4 hours in Williams E media, supplemented with 5% FBS, 1% Penicillin-Streptomycin, 10 nM insulin, and 10 nM dexamethasone.

#### Tissue harvest and tumor analysis

Tumor burden for each liver was determined separately by two individuals blinded to genotype. Mice were euthanized in the ad-lib fed state between 0900 and 1200 and livers were immediately harvested and weighed. Next, each liver lobe was grossly dissected and examined for visible tumors, which were counted and measured in diameter using calipers. A section of the left lateral lobe was fixed in 10% neutral-buffered formalin for paraffin-embedding. The remaining liver was divided into normal adjacent and paired tumor tissue, snap-frozen in liquid nitrogen and stored at −80°C until further biochemical analysis.

#### Plasma analysis

Tail vein blood was collected using capillary tubes. Tubes were centrifuged at 3,000 × *g* for 15 minutes and the supernatant was collected. Prior to analysis, this plasma was diluted to 1:3 with 0.9% saline. Samples were analyzed for Aspartate aminotransferase (AST) activity and Alanine aminotransferase (ALT) activity following the manufacturers’ protocols.

#### Immunohistochemistry

Formalin-fixed tumor samples were processed and embedded in paraffin. Tissue sections (4 μm) were deparaffinized and stained with Ki67 and Cleaved Caspase-3. Immunohistochemistry was visualized using DAKO EnVision + Dual Link System-HRP (DAB+) for 5 minutes. Tumor slides were imaged using an Olympus BX-61 light microscope while blinded to genotype. Three separate high-power field images/slide/mouse were imaged and analyzed using ImageJ. The positive nuclei fraction was determined using: threshold particle analysis. Threshold particle analysis identifies the number of both total nuclei and IHC-reactive nuclei by colorimetric threshold. The ratio of IHC-reactive/total nuclei was defined as % positive.

#### Western Blots

Snap-frozen liver tissue was homogenized in a buffer containing 40 mM HEPES, 120 mM NaCl, 50 mM NaF, 5 mM Sodium Pyrophosphate decahydrate, 5 mM β-glycerolphosphate, 1 mM EDTA, 1 mM EGTA, 10% Glycerol (v/v), 1% Igepal CA-630 (v/v), with 1X Protease Arrest protease inhibitor and 1 mM DTT. Homogenates were incubated at 4°C for 30 minutes and centrifugation at 21,000 × *g* before the supernatants were collected. Proteins were separated by 10% Tricine-SDS-PAGE gel, transferred to 0.22 μM nitrocellulose membranes, and blocked with TBST (50 mM Tris, 150 mM NaCl, and 0.05% Tween20) supplemented with 5% nonfat dry milk. Blocked membranes were incubated with primary antibodies at 4°C overnight and the following day fluorescent secondary antibodies for 1 hour at RT. Finally, immuno detected proteins were visualized using the Li-Cor Odyssey CLx system.

#### Quantitative PCR (qPCR)

Total RNA from liver tissue was extracted using TRIzol method. cDNA synthesis of equal amounts of RNA from each sample was achieved using the High-Capacity cDNA Reverse Transcription Kit. SYBR Green ER SuperMix was used during qPCR. Relative abundance of mRNA was normalized to the abundance of u36B4 mRNA.

#### 8-hydroxy-2-deoxyguanosine (8-OHdG) ELISA Assay

15 day old constitutive, hepatocyte-specific Mpc1 knockout (MPC LivKO) and wild-type (WT) mice were injected with either 1 mg/kg of DEN or PBS. 24 hours after injection mice were euthanized, and their livers were flash frozen in liquid nitrogen. DNA was isolated from frozen liver tissue using a QIAGEN DNeasy Kit. DNA was digested by the endonuclease Benzonase for 1 hour at 37°C followed by a 30 minute treatment with alkaline phosphatease at 37°C. Samples were diluted to a DNA concentration of 100 ng/μl. From there, 5 μg of DNA was added per well and DNA/RNA Oxidative Damage ELISA Kit instructions were followed.

#### Transcriptomic sequencing

Total RNA was collected from tumor and paired normal-adjacent liver samples using the QIAGEN miRNeasy kit. RNA from four samples each of wild-type tumor (WT-Tumor), paired wild-type normal-adjacent (WT normal adjacent), MPC LivKO tumor (MPC LivKO-Tumor), and paired MPC LivKO normal-adjacent (MPC LivKO normal adjacent) tissue was isolated. Each tumor and its paired normal-adjacent tissue were analyzed in a paired manner. Library preparation and sequencing were performed using the Illumina mRNA-Seq workflow. Initial mapping was done using the HISAT2 software program. The resulting BAM files were analyzed using the RNaseq pipeline in the Partek Genomics Suite software package. For data normalization, the raw number of reads for each transcript was converted to Fragments Per Kilobase of transcript per Million mapped reads (FPKM). FPKM values were log transformed and unsupervised clustering was performed on samples based on normalized expression of genes with variation in Euclidean distance among samples of at least 2.5 standard deviations using Cluster 3 software. Heatmaps were generated using Java TreeView software. Pathway analysis was conducted using the QIAGEN Ingenuity Pathway Analysis software program.

#### DHE Assay

10 mm sections of OCT-frozen liver tissue were cut and placed on the same slide. Tissue sections were stained with 10 mM DHE for 15 minutes in PBS and imaged using an Olympus FLUOVIEW FV1000 confocal microscope. Sections were co-treated with 10 mM antimycin A during DHE staining as positive control. Mean fluorescence intensity of at least 200 cell nuclei was determined for each image and normalized to nuclei from untreated normal tissue.

#### Glutathione Assay

Tumor and normal adjacent tissue samples were homogenized in 5% SSA (5-Sulfosalicylic acid) buffer at the time of sacrifice. The samples were centrifuged to remove precipitated proteins. Supernatants were assayed for total glutathione (GSH) content according to the Sigma Glutathione Assay Kit method. Glutathione disulfide (GSSG) was measured after reacting the sample for 2 hours with 20 μl of a 1:1 mixture of 2-vinylpyridine and ethanol per 100 μl of sample to mask GSH. GSSG was measured GSH-depleted samples using using the Sigma Glutathione Assay Kit. Glutathione determinations were normalized to protein by solubilizing SSA precipitates in 0.1 N NaOH and measuring protein content using the BCA Assay Kit.

#### AlamarBlue Assay

Hepa1–6 cells were treated with BSO (L-Buthionine-sulfoximine), UK5099, and N-acetyl-cysteine (NAC) for 48 hours. Viability was assessed by resazurin cell viability assay ex/em 560nm/590nm. In short, 25,000 cells were plated per well of a standard 96-well plate. Media was repalced with 200 μL of media containing corresponding treatment conditions and allowed to grow for 48 hours. After 48 hours of growth, 20 μL of 0.15 mg/ml resazsurin was added per well, incubated for 3 hours, and then fluorescence was measured.

#### Crystal Violet Cell Cytotoxicity Assay

Hepa 1–6 cells (7500 cells/well) were seeded on a clear, 96-well plate. After 18 hours, cells were treated with 200 μl of media containing DMSO, 5 μM UK5099, 1 mM BSO, 1 mM BSO + UK5099, 5 mM NAC, 1 mM BSO + 5 mM NAC, or 1 mM BSO + 5mM NAC + 5 μM UK5099. Media containing treatment was changed every 24 hours until cells had grown for 48. A live/dead assay was then performed using a the Crystal Violet Cell Cytotoxicty Assay Kit. Cells were washed with 200 μl of 1x washing solution and incubated in 50 μl of crystal violet staining solution for 20 minutes. Cells were washed four times with 200 μl of 1x washing solution to remove dead cells. Cells were then incubated in 100 μl of solubilization solution for 20 minutes, and absorbance was measured at 595 nm.

#### Clonogenic Assay

1.5 × 10^5^ Hepa1–6 cells were plated in 60 mm dishes for 48 hours and treated in DMEM supplemented with 10% FBS, 1% sodium pyruvate, and 1% Pen Strep with 1 mM BSO, 5 μM UK5099, and 10 mM NAC for 72 hours prior to clonogenic assay. Cells were detached using 0.25% trypsin, combined with floating cells, and pelleted via centrifugation at 200 × *g* for 5 minutes. Pellets were resuspended in fresh complete DMEM media and the total cell population was counted using a Beckman Coulter Counter. 500–20,000 cells per dish for clonogenic survival were then plated in 60-mm dishes that had been previously plated one day prior with a feeder layer of 100,000 immortalized chinese hamster ovary cells that had been lethally irradiated with 30 Gy of 250 kVp X-rays. Clones were grown for 8 days in complete media, fixed with 70% ethanol, stained with Coomassie blue, and colonies greater than 50 cells were counted. The plating efficiencies of treatment groups for each cell line were normalized to the control groups. Feeder layer alone plates were included in every experiment to ensure that all the feeder cells had been clonogenically inactivated. The survival analysis was performed using a minimum of two cloning dishes per experimental condition, and the experiment were repeated a minimum of three times.

#### Monochlorobimane Assay

Hepatocytes were washed with PBS and DMEM media containing 5 mM glucose and 50 μM monochlorobimane or 5 mM glucose plus 5 mM glutamine and 50 μM monochlorobimane was added. Immediately after media addition cells were analyzled (ex/em 370/490) on a 90 s interval for 40 minutes.

#### In-vivo stable isotope tracer measurement

Mpc1 LivKO (AAV-Cre) and wild-type (AAV-EV) mice were retro-orbitally injected with a solution of 200mM (U)^13^C glutamine at a dosage of 400 mg/kg whole body mass. Two injections were administered 15 minutes apart. 30 minutes after the first injection, mice were anesthetized with isoflurane and the left lateral lobe of the liver was rapidly frozen using a freeze-clamp apparatus. Frozen lobules of liver were lyophilized overnight. Lyophilized tissue was disrupted using a bead mill homogenizer in ice cold 4.5:4.5:1 methanol/acetonitrile/H_2_O to extract metabolites. Homogenate was incubated at −20°C for 1 hour followed by a 10 minute centrifugation at maximum speed. Supernatants were transferred to fresh tubes and evaporated using a speed-vac. The resulting dried metabolite extracts were derivatized using methyoxyamine hydrochloride (MOC) and N,O-Bis(trimethylsilyl)trifluoroacetamide (TMS). Briefly, dried extracts were reconstituted in 60 μL of 11.4 mg/ml MOC in anhydrous pyridine, vortexed for 5 min, and heated for 1 hour at 60°C. Next, 40 μL TMS was added to each sample, and samples were vortexed for 1 minute before heating for 30 minutes at 60°C. Samples were then immediately analyzed using GC/MS. GC chromatographic separation was conducted on a Thermo Trace 1300 GC with a TraceGold TG-5SilMS column. 1 μL of derivatized sample was injected into the GC operating in split mode (split ratio: 20–1; split flow: 24 μL/min, purge flow: 5 mL/min, Carrier mode: Constant Flow, Carrier flow rate: 1.2 mL/min). The GC oven method was as follows: 80°C for 3 minutes, ramp to 280°C at a rate of 20°C/minute to a maximum temperature of 280°C, and the oven was held at 280°C for 8 minutes. The injection syringe was washed 3 times with methanol and 3 times with pyridine between each sample. Metabolites were detected using a Thermo Q-Exactive Plus or Thermo ISQ 7000 mass spectrometers. The Q-Exactive Plus mass spectrometer was operated from 3.90 to 21.00 minutes in EI mode (−70eV) in full scan (56.7–850 m/z) at 60K resolution and the ISQ 7000 mass spectrometer was operated in SIM mode for glucose molecular ion isotopologues (m/z: M+0 = 554, M+1 = 555, M+2 = 556, M+3 = 557, M+3 = 558, M+4 = 559, M+5 = 560). XCalibur was used for istopologue peak identification and integration. Metabolite profiling data was analyzed using Tracefinder 4.1 utilizing standard verified peaks and retention times.

#### Primary Hepatocyte Tracer Measurement

Hepatocytes were washed with PBS and media was replaced with DMEM containing 5 mM glucose, 5 mM glutamine, and 50 μM monochlorobimane for 40 minutes to deplete cellular GSH levels. Hepatocytes were washed and media was replaced with DMEM containing 5 mM glucose and 5 mM (U)^13^C glutamine and DMSO or the specific MPC-inhibitor, 5 μM UK5099, for 1 hour. Following the incubation, hepatocytes were washed 2 times with ice-cold PBS, 2 times with ice-cold distilled water, and then immediately frozen in liquid nitrogen. Frozen plates of hepatocytes were lyophilized overnight and extracted using cold (−20°C) 4.5:4.5:1 methanol/acetonitrile/H_2_O by 2 cycles of vortexing, freeze-thawing, and water bath sonication. The samples were then incubated at −20°C for 1 hour followed by a 10 minutes centrifugation at maximum speed. Supernatants were transferred to fresh tubes and evaporated using a speed-vac. Samples were resuspended in 60 μL of 1:1 acetonitrile:water, sonicated for 20 minutes, vortexed for 5 minutes, incubated for 1 hour at −20°C, and centrifuged for 10 minutes at max speed to remove any protein carry over. Supernatants were collected and transferred to autosampler vials. A Phenomenex Luna aminopropyl column attached to a Thermo Vanquish Flex UHPLC separated metabolites using a HILIC mobile phase system (A:20mM NH_4_-Acetate and 20mM NH_4_OH; B:90:10 acetonitrile:water). LC flow was 0.2 mL/min. The gradient was as follows: 85% B, linear decrease to 0% B by 34 min, and holding at 0% B from 34–38 minutes. The mass spectrometer operated in negative mode. Targeted selected ion monitoring (tSIM) was used to isolate GSSG isotopologues (tSIM: 610.1447–622.1447 m/z; M+0 = 611.1451 m/z, M+1 = 612.1485 m/z, M+2 = 613.1518 m/z, M+3 = 614.1552 m/z, M+4 = 615.1585 m/z, M+5 = 616.1619 m/z) and GSH isotopologues (tSIM: 305.0765–313.0765 m/z; M+0 = 306.0765 m/z, M+1 = 307.0799 m/z, M+2 = 308.0832 m/z, M+3 = 309.0866 m/z, M+4 = 310.0899 m/z, M+5 = 311.0933 m/z). XCalibur was used for istopologue peak identification and integration.

#### Principle Component Analysis

Principle Component Analysis (PCA) score plot of the first and second principle components (PCs) generated from a dataset of 86 metabolites extracted from MPC LivKO and WT liver tissue. The website ClustVis (https://biit.cs.ut.ee/clustvis/) was used to generate the PCA plot. ClustVis utilizes R code (https://www.r-project.org) for generation of PCA plots.

### QUANTIFICATION AND STATISTICAL ANALYSIS

As indicated within figure legends the Student’s t test was used when comparing the means of two groups, and one- and two-way analysis of variance (ANOVA) with Tukey’s post hoc testing was used when comparing the means of more than two groups. A limited number of statistical outliers were excluded after identification by the Grubbs test. P values less than 0.05 were considered statistically significant (*p < 0.05, **p < 0.01, ***p < 0.001). Data were organized and analyzed using Microsoft Excel, SigmaPlot, and GraphPad Prism software. Sample size (n) and definition of “n” are found in figure legends.

### DATA AND CODE AVAILABILITY

Transcriptomic data generated during this study are available at the Gene Expression Omnibus database: GSE132728 (https://www.ncbi.nlm.nih.gov/geo/query/acc.cgi?acc=GSE132728).

## Supplementary Material

1

2

3

4

5

## Figures and Tables

**KEY RESOURCES TABLE:** 

REAGENT or RESOURCE Antibodies	SOURCE	IDENTIFIER
Mpc1	Cell Signaling	Cat#14462; RRID: AB_2773729
Mpc2	Cell Signaling	Cat#46141; RRID: AB_2799295
VDAC	Cell Signaling	Cat#4664; RRID: AB_10557420
Cleaved Caspase-3	Cell Signaling	Cat#9661; RRID: AB_2341188
Ki67	Abcam	ab#16667; RRID: AB_302459
Goat anti-Rabbit DyLight 800	ThermoFisher	SA5-35571; RRID: AB_2556775
Goat anti-Mouse DyLight 800	ThermoFisher	SA5-10176; RRID: AB_255675
DAKO EnVision + Dual Link System-HRP (DAB+)	Dako	K4065
Bacterial and Virus Strains		
AAV8.TBG.PI.Cre.rBG	UPenn Viral Vector Core	AV-8-PV1090
AAV8.TBG.PI.Null.bGH	UPenn Viral Vector Core	AV-8-PV0148
Chemicals, Peptides, and Recombinant Proteins		
Penicillin-Streptomycin	ThermoFisher	15140-122
Insulin	Sigma	I9278
Dexamethasone	Sigma	D4902
Capillary blood tubes	Sarstedt	16.444.100
Fetal Bovine Serum	Atlanta Biologicals	S11150
GlutaMAX	GIBCO	3505006
Protease Arrest	G Biosciences	786-437
Igepal CA-630	Sigma	I8896
N-nitrosodiethylamine (DEN)	Sigma	N0258
Trizol	Life Technologies	15596018
High-Capacity cDNA Reverse Transcription Kit	Applied Biosystems	4368814
SYBR Green ER SuperMix	Life Technologies	11760-100
DNeasy Kit	QIAGEN	#69504
Benzonase	Sigma	E1014
Alkaline Phosphatase	ThermoFisher	EF0654
miRNeasy	QIAGEN	#217004
Sulfosalicylic acid	Sigma	90275
Pierce BCA Assay Kit	ThermoFisher	23225
2-vinylpyridine	Sigma	132292
Resazurin	Sigma	R7017
UK5099	Tocris	4186
L-Buthionine-sulfoximine	Sigma	B2515
N-acetyl cysteine	Sigma	A9165
Monochlorobimane	Sigma	69899
U-^13^C Glutamine	Cambridge Isotopes	CLM-1822
Methyoxyamine hydrochloride (MOC)	Sigma	226904
N,O-Bis(trimethylsilyl)trifluoroacetamide (TMS)	Sigma	155195
Carbon Tetrachloride (CCl_4_)	Sigma	289116
Dihydroethidium	ThermoFisher	D1168
Williams E Media	ThermoFisher	12551-032
Critical Commercial Assays		
Glutathione Assay Kit	Sigma	CS0260
DNA/RNA Oxidative Damage ELISA Kit	Caymen Chemical	589320
ALT Activity Assay	ThermoScientific	TR18503
AST Activity Assay	ThermoScientific	TR70121
Crystal Violet Cell Cytotoxicity Assay Kit	BioVision	K329-1000
Deposited Data		
RNA-sequencing of paired normal-adjacent and liver tumor tissue from WT and Liver MPC-knockout mice	This paper	GEO: GSE132728
Tissue Cancer Genome Atlas	http://www.cbioportal.org/	N/A
Experimental Models: Cell Lines		
Hepa1-6	Sigma	92110305
Experimental Models: Organisms/Strains		
Mouse: WT: Mpc1^fl/fl^: C57Bl6/J-Mpc1^fl/fl^	Gray et al., 2015	N/A
Mouse: MPC LivKO: Mpc1^fl/fl^: C57Bl6/J-Mpc1^fl/fl^+Alb^Cre^	Gray et al., 2015	N/A
Oligonucleotides		
Mpc1 Forward Primer: AACTACGAGATGAGTAAGCGGC	IDT	N/A
Mpc1 Reverse Primer: GTGTTTTCCCTTCAGCACGAC	IDT	N/A
Mpc2 Forward Primer: CCGCTTTACAACCACCCGGCA	IDT	N/A
Mpc2 Reverse Primer: CAGCACACACCAATCCCCATTTCA	IDT	N/A
36b4 Forward Primer: CGTCCTCGTTGGAGTGACA	IDT	N/A
36b4 Reverse Primer: CGGTGCGTCAGGGATTG	IDT	N/A
Software and Algorithms		
Excel	Microsoft	N/A
SigmaPlot	Systat Software	N/A
GraphPad Prism	Graphpad Software Inc.	N/A
HiSAT2	https://ccb.jhu.edu/software/hisat2/index.shtml	N/A
Partek Genomics Suite	Partek Inc.	N/A
Ingenuity Pathway Analysis	QIAGEN	N/A
Cluster 3	http://bonsai.hgc.jp/~mdehoon/software/cluster/software.htm	N/A
Java TreeView	http://jtreeview.sourceforge.net	N/A
ClustVis	https://biit.cs.ut.ee/clustvis/	N/A
Other		
Luna aminopropyl LC column	Luna aminopropyl LC column	Luna aminopropyl LC column

## Highlights

- The MPC is retained in HCC, supporting TCA cycle pyruvate metabolism
- MPC disruption directs glutamine to the TCA cycle away from glutathione synthesis
- Glutathione synthesis is diminished, impairing hepatocellular tumorigenesis
