# Supplementary material for: Disrupting Mitochondrial Pyruvate Uptake Directs Glutamine into the TCA Cycle away from Glutathione Synthesis and Impairs Hepatocellular Tumorigenesis
Source: Cell Rep. Author manuscript; Available in PMC 2019 Sep 16. (PMC6746334; doi:10.1016/j.celrep.2019.07.098)
Supplement: 1 [file NIHMS1539126-supplement-1.pdf]

Cell Reports, Volume *28*

Supplemental Information

Disrupting Mitochondrial Pyruvate Uptake Directs Glutamine into the TCA Cycle away from Glutathione Synthesis and Impairs Hepatocellular Tumorigenesis

Sean C. Tompkins, Ryan D. Sheldon, Adam J. Rauckhorst, Maria F. Noterman, Shane R. Solst, Jane L. Buchanan, Kranti A. Mapuskar, Alvin D. Pewa, Lawrence R. Gray, Lalita Oonthonpan, Arpit Sharma, Diego A. Scerbo, Adam J. Dupuy, Douglas R. Spitz, and Eric B. Taylor

Figure S1, related to Figure 2

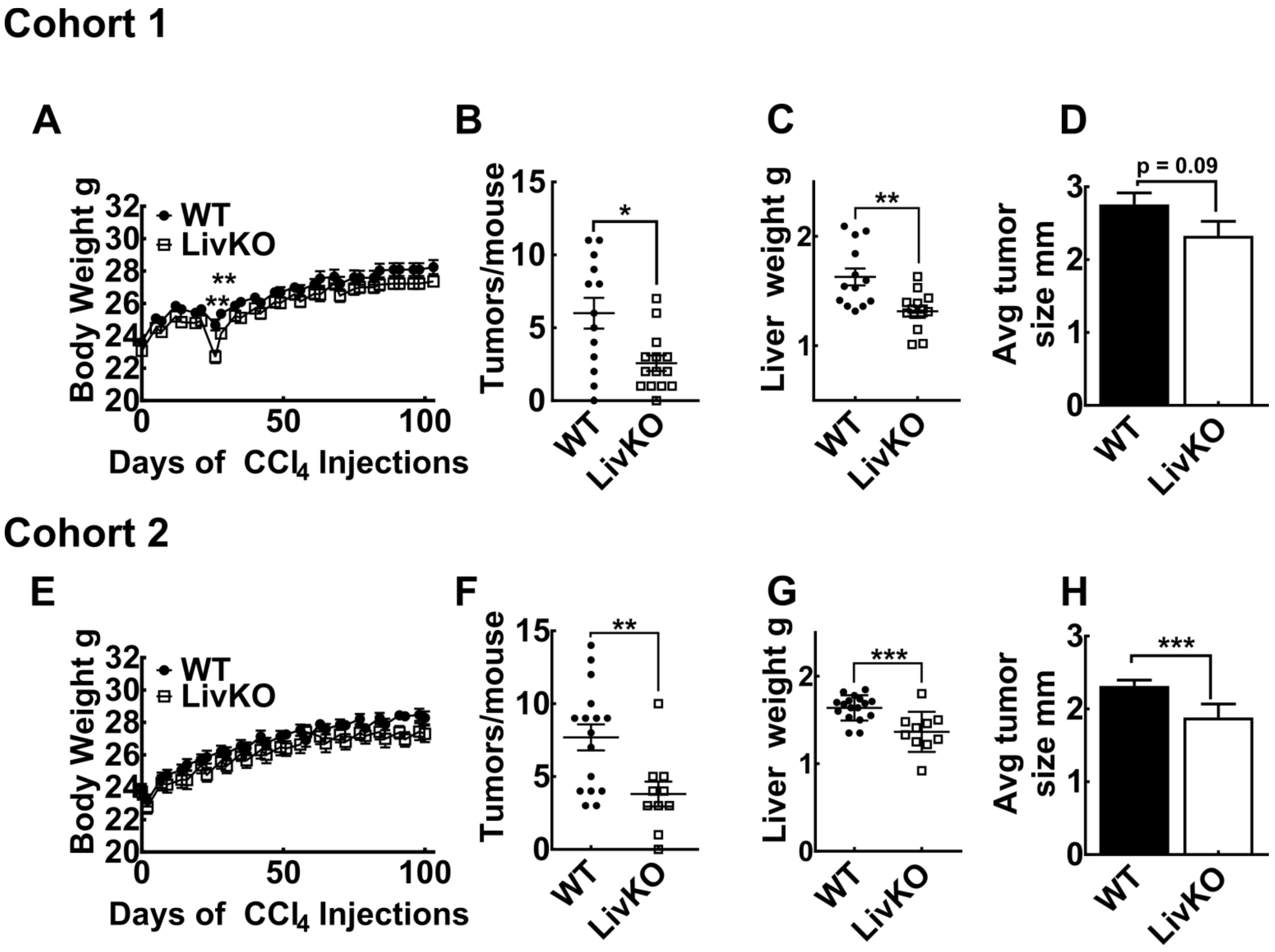

Figure S1, related to Figure 2.

(A-C) Cohort 1 MPC LivKO and WT mice body weight over course of injections (A), gross liver tumor burden (B), and gross liver weight (C), n=13-15 biological replicates.

(D) Cohort 1 average tumor size as measured by calipers at time of sacrifice WT tumors n=37, LivKO tumors n=25. Note, for Cohort 1, not all animals had their tumors measured by calipers.

(E-G) Cohort 2 MPC LivKO and WT mice body weight over course of injections (E), gross liver tumor burden (F), and gross liver weight (G), n=11-16 biological replicates.

(H) Cohort 2 average tumor size as measured by calipers at time of sacrifice WT tumors n=115, LivKO tumors n= 29. Note, for Cohort 2, all animals had their tumors measured by calipers.

Data are presented as mean ± SEM (*p<0.05, **p<0.01, ***p<0.001).

Figure S2, related to Figure 2

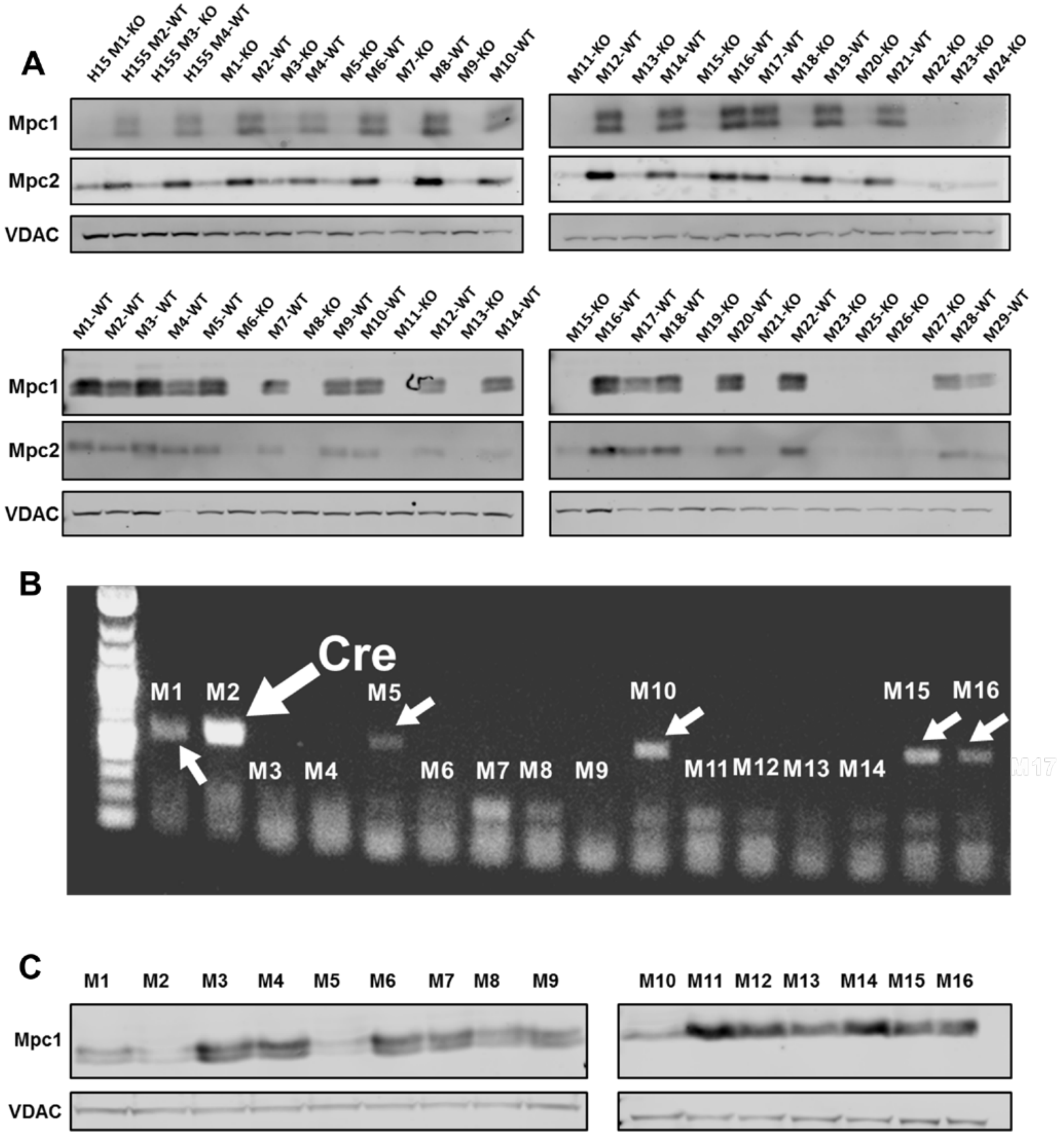

Figure S2, related to Figure 2.
(A) Western blot for Mpc1 and Mpc2 in normal-adjacent liver tissue from both hepatic carcinogenesis cohorts. VDAC control.
(B) PCR genotyping of livers from MPC LivKO and WT mice used in Figure 2I. Presence of a Cre band at 565 base pairs denotes MPC LivKO genotype. Image mode converted from RBG to greyscale.
(C) Western blot of livers mice used in Figure 2I for Mpc1. Cre is present and active in MPC LivKO mice at 16 days of age, however the deletion is only partial in the developing mouse liver. VDAC control.

Figure S3, related to Figure 2

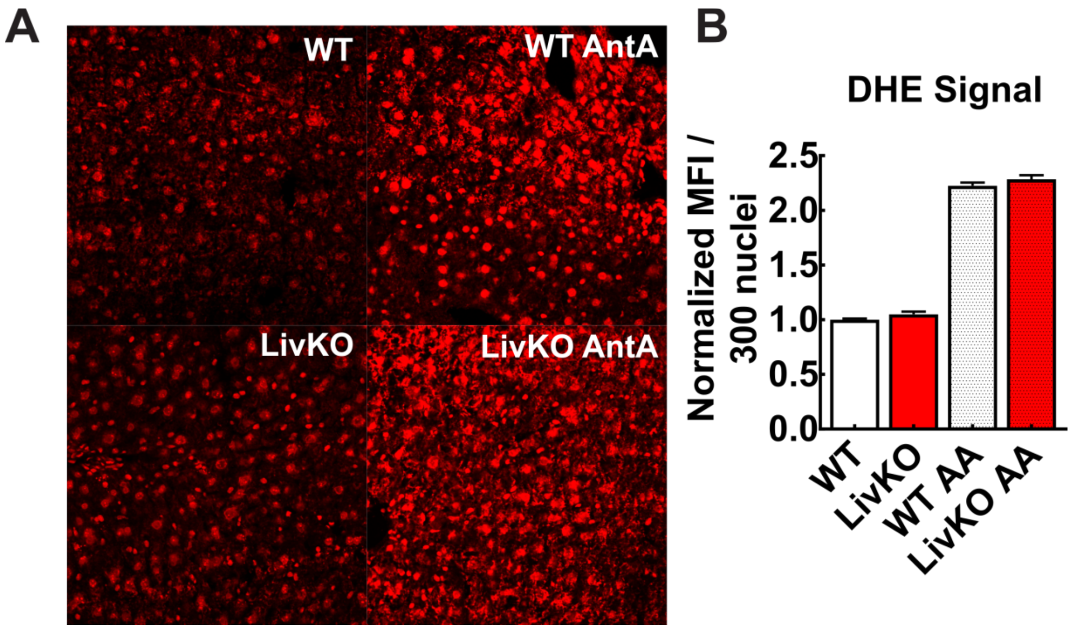

Figure S3, related to Figure 2.
A) 10 mM sections of WT or MPC LivKO liver were stained with 10 mM dihydroethidium (DHE) on the same slide for 30 min prior to imaging by confocal microscopy. For a positive control, tissue sections were incubated with 10 mM antimycin A during DHE staining.
(B) The mean fluorescence intensity (MFI) of 200 nuclei from six randomly selected areas quantified using ImageJ software, n=4 biological replicates.
Data are presented as mean ± SEM (*p<0.05, **p<0.01, ***p<0.001).

Figure S4, related to Figure 4

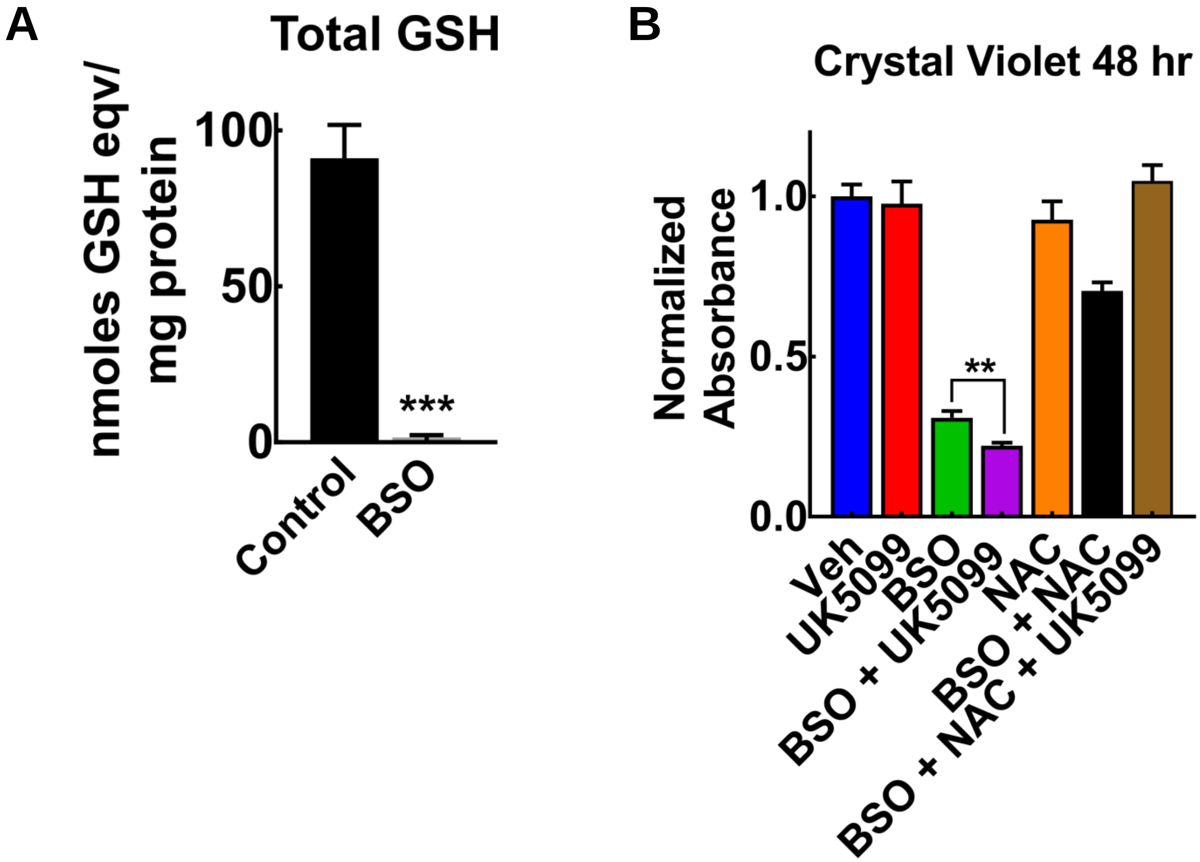

Figure S4, related to Figure 4.
(A) Total glutathione (GSH) levels in Hepa1-6 cells treated with 1 mM BSO for 48 hours, n=4 replicate wells.
(B) Hepa1-6 cells treated for 48 hours with vehicle, 1 mM buthionine sulfoximine (BSO), 5 µM UK5099 (UK), 1 mM BSO + 5 µM UK5099, 5 mM N-acetyl cysteine (NAC), 1 mM BSO + 5 mM NAC, or 1 mM BSO + 5 µM UK5099 + 5 mM NAC. Viability measured by crystal violet assay, n=8 replicate wells.
data are presented as mean ± SEM (*p<0.05, **p<0.01, ***p<0.001).

Figure S5, related to Figure 5

A

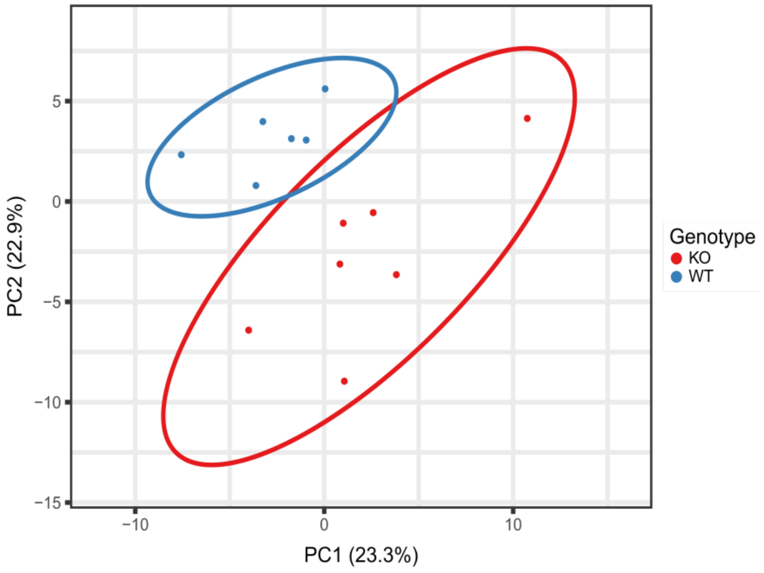

B

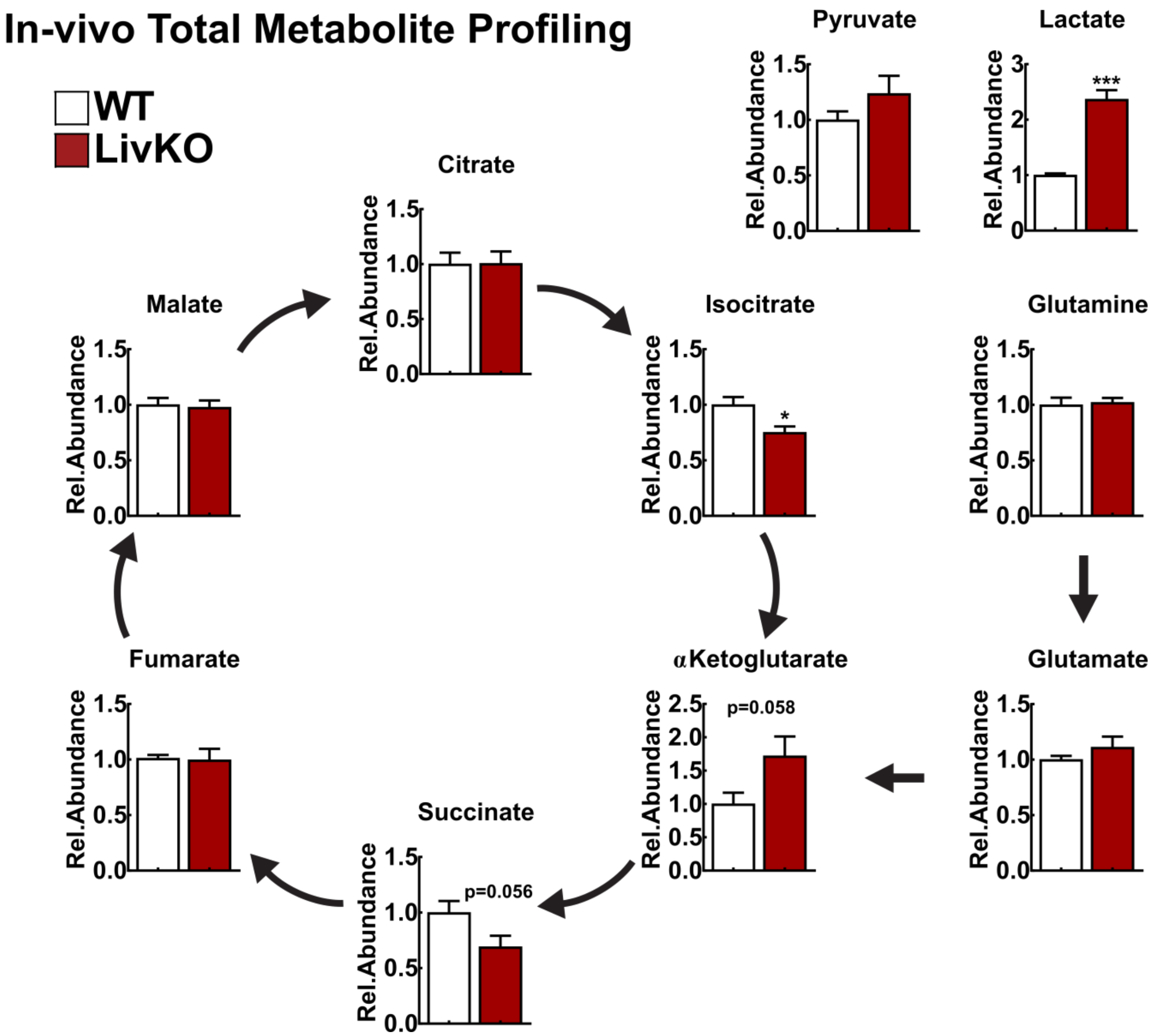

Figure S5, related to Figure 5.

(A) Principle Component Analysis (PCA) score plot of the first and second principle components (PCs) generated from 86 metabolites extracted from MPC LivKO and WT liver tissue. PCA demonstrates clear separation between MPC LivKO and WT. PC1 explains 23.3% of chemical variance while PC2 explains 22.9%. From the PCA data two separate groups were observed corresponding to WT and MPC LivKO mice. Each dot represents an individual animal. PCA plot created using ClustVis (https://biit.cs.ut.ee/clustvis/). n=6-7 biological replicates.

(B) Relative abundance of TCA cycle metabolites, glutamate, glutamine, pyruvate, and lactate in livers of MPC LivKO and WT mice after injection of (U)^{13}C- glutamine, n=6-7 biological replicates. Note, the glutamine, glutamate, and αketoglutarate panels are shown in Figure 5.

Data are presented as mean ± SEM (*p<0.05, **p<0.01, ***p<0.001).

Figure S6, related to Figure 6

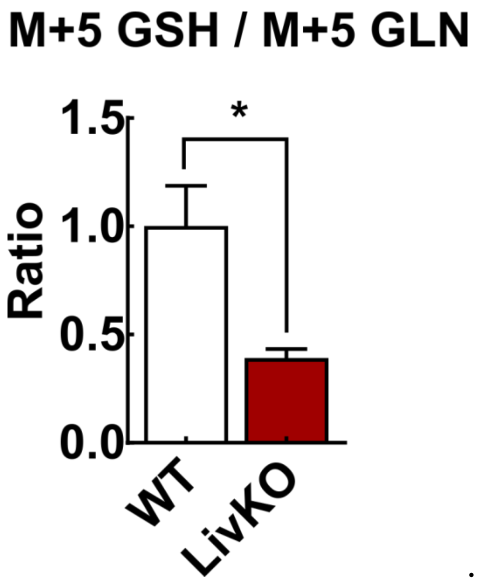

Figure S6, related to Figure 6.
Ratio of M+5 glutathione to M+5 glutamine signal in MPC LivKO or WT primary hepatocytes, n=4 biological replicates. Data are presented as mean ± SEM (* $p<0.05$, ** $p<0.01$, *** $p<0.001$).
